# Melatonin Improves the Resistance of Oxidative Stress-Induced Cellular Senescence in Osteoporotic Bone Marrow Mesenchymal Stem Cells

**DOI:** 10.1155/2022/7420726

**Published:** 2022-01-18

**Authors:** Weikai Chen, Nanning Lv, Hao Liu, Chao Gu, Xinfeng Zhou, Wanjin Qin, Angela Carley Chen, Liang Chen, Huilin Yang, Xi Chen, Tao Liu, Fan He

**Affiliations:** ^1^Department of Orthopaedics, The First Affiliated Hospital of Soochow University, Suzhou 215006, China; ^2^Department of Orthopaedics, The Second People's Hospital of Lianyungang, Lianyungang, Jiangsu 222003, China; ^3^Orthopaedic Institute, Medical College, Soochow University, Suzhou 215000, China; ^4^School of Public Health and Health Systems, University of Waterloo, Waterloo, Ontario, Canada N2L 3G1; ^5^Department of Pathology, The Third Affiliated Hospital of Soochow University, Changzhou 213003, China

## Abstract

Accumulation of senescent bone marrow-derived mesenchymal stem cells (BMMSCs) has led to an age-related bone loss. However, the role of stem cell senescence in estrogen deficiency-induced osteoporosis remains elusive. Though melatonin plays a vital role in bone metabolism regulation, the underlying mechanisms of melatonin-mediated antiosteoporosis are partially elucidated. Therefore, this study purposed to explore (1) whether estrogen deficiency causes cellular senescence of BMMSCs, and if so, (2) the potential of melatonin in preventing bone loss via senescence signaling inhibition. BMMSCs derived from ovariectomized (OVX) rats (OVX BMMSCs) showed an impaired osteogenic capacity, albeit having comparable levels of senescence biomarkers than the sham cells. When exposed to low levels of hydrogen peroxide (H_2_O_2_), OVX BMMSCs rapidly exhibited senescence-associated phenotypes such as the increased activity of senescence-associated *β*-galactosidase (SA-*β*-gal) and upregulation of cell cycle inhibitors. Notably, the *in vitro* treatment with melatonin hindered H_2_O_2_-induced senescence in OVX BMMSCs and restored their osteogenic capacity. Treatment with either SIRT1 inhibitor (sirtinol) or melatonin receptor antagonists (luzindole and 4-P-PDOT) eliminated melatonin protective effects, thus indicating its potential in preventing stem cell senescence via SIRT1 activation through the melatonin membrane receptors. Following *in vivo* intravenous administration with melatonin, it successfully protected the bone microstructure and preserved the antisenescence property of BMMSCs in OVX rats. Collectively, our findings demonstrated that melatonin protected against estrogen deficiency-related bone loss by improving the resistance of BMMSCs to cellular senescence. Therefore, melatonin-mediated antisenescence effect on stem cells provides vital information to facilitate the development of a novel and effective strategy for treating postmenopausal OP.

## 1. Introduction

Estrogen deficiency is a major cause of postmenopausal osteoporosis (OP) that leads to an imbalance in osteoblast-mediated bone formation and osteoclast-mediated bone resorption [1]. Bone marrow-derived mesenchymal stem cells (BMMSCs), the progenitor cells of osteoblasts, play a crucial role in mediating bone homeostasis. Based on emerging evidence, BMMSCs derived from OP patients exhibit degenerative properties such as a decreased proliferative capacity, impaired ability to migrate, and preference for adipogenic differentiation [2]. The senescent stem cells accumulated in the bone marrow with age are considered to be responsible for the age-related bone loss [3].

Cellular senescence is characterized by an irreversible proliferation arrest with distinctive phenotypic alterations such as enlarged cell sizes, resistance to apoptosis, permanent cell cycle arrest, and increased senescence-associated *β*-galactosidase (SA-*β*-gal) [4]. Besides, various types of stress can induce cellular senescence, such as telomere shortening, DNA damage, excessive reactive oxygen species (ROS), and oncogenic mutations [5]. The cell cycle-regulating factors involved in the senescence process include P53, P21^Cip1/Waf1^ (P21), and P16^Ink4*α*^ (P16). Specifically, the accumulation of P16 triggers the onset of cellular senescence [6], while the elimination of *P16*-positive senescent cells in aged mice potentially promotes bone formation [7]. However, the role of stem cell senescence in estrogen deficiency-induced OP remains controversial. A study by Wu et al. reported that the BMMSCs from ovariectomized (OVX) rats had an increase in senescence biomarkers such as P53 and P16 [8]. Elsewhere, an emerging study showed contrary findings that elimination of senescent cells failed to rescue bone loss in OVX mice, thus indicating an independent role of cellular senescence in estrogen deficiency-induced OP [9].

Melatonin, mainly produced by the pineal gland, has been shown to play an important role in the regulation of bone metabolism [10]. Interestingly, during MSC osteogenesis, melatonin enhanced the expression of alkaline phosphatase (ALP) and matrix mineralization, even in the presence of proinflammatory cytokines [11]. Briefly, the intracellular signaling is transmitted via two high-affinity G protein-coupled receptors, MT1 and MT2 that are widely distributed in multiple tissues and organs [12]. MT1 receptors mediate the inhibition effect of melatonin on human breast cancer cells [13], whereas MT2 receptors are associated with osteoblast differentiation via the extracellular signal-regulated kinase (ERK)1/2 signaling cascade [14]. According to reports, oral administration of melatonin was able to effectively cure bone loss in OVX mice [15] and improve bone mineral density (BMD) at the femoral neck in postmenopausal women without major side effects [16].

Increased ROS with a simultaneous decrease in antioxidant enzymes were reported in OVX rats and postmenopausal women [17, 18]. Oxidative stress induced by ROS generation and detoxication imbalance is a crucial factor in cellular senescence [19]. Overaccumulation of hydrogen peroxide (H_2_O_2_) induces cellular senescence, therefore resulting to the decline in osteogenic differentiation of human MSCs [20]. Notably, melatonin protects MSCs against H_2_O_2_-induced senescence by upregulating the silent information of regulator type 1 (SIRT1), an important gene controlling cellular longevity [21]. In this study, we hypothesized that melatonin potentially ameliorated estrogen deficiency-induced bone loss by preventing senescence of BMMSCs derived from OVX rats (OVX BMMSCs). Therefore, we exposed BMMSCs to sublethal concentrations of H_2_O_2_ to induce senescence before evaluating the protective effects of melatonin. Subsequently, we injected OVX rats with melatonin via the tail vein and analyzed the antisenescence properties of BMMSCs.

## 2. Material and Methods

### 2.1. Animals

Eight-week-old female Sprague–Dawley (SD) rats (180 ± 13 g) were purchased from the Animal Center of Soochow University. The ovariectomy was performed on the rats following the standard method as previously described [22]. Bilateral OVX was carried out by the abdominal approach before excising ovaries from both sides of rats anesthetized with pentobarbital (30 mg/kg body weight, intraperitoneally; Yuanye, Shanghai, China). Similar procedures were followed in the sham group except ovaries were left intact. After the surgeries, the rats were sutured and injected with penicillin for three days (80,000 Units/rat, intramuscularly; Yuanye). Rats were housed under specific pathogen-free conditions with free access to water and fed on a standard laboratory rodent diet *ad libitum*. Animal experiments were performed following their approval by the Ethics Committee of Soochow University.

### 2.2. Isolation and Culture of BMMSCs

Bone marrow cells were flushed out from the tibiae and femurs of OVX- or sham-operated rats, using alpha minimum essential medium (*α*-MEM, Thermo Fisher Scientific, Waltham, MA). Red blood cells were then removed using red blood cell lysis buffer (Beyotime Institute of Biotechnology, Haimen, China). After washing, bone marrow cells were incubated in a 75 cm^2^ culture flask (Costar, Tewksbury, MA) containing *α*-MEM supplemented with 10% fetal bovine serum (FBS), 100 U/mL penicillin, and 100 *μ*g/mL streptomycin (all from Thermo Fisher Scientific) and the cultures maintained at 37°C with 5% CO_2_ for 3 days. The medium was changed, to remove nonadherent cells, and the adherent cells cultured in fresh medium. After attaining 80% confluence, 0.25% trypsin (Thermo Fisher Scientific) was added and the primary rat BMMSCs were replated. Subsequent experiments used cells that were from passage one.

### 2.3. Treatments with H_2_O_2_, Melatonin, Compound C, Luzindole, 4-P-PDOT, and Sirtinol

To induce premature senescence, BMMSCs at approximately 50% confluence were exposed to H_2_O_2_ for 2 h (Sigma-Aldrich, St. Louis, MO) and then cultured for an additional 3 days. Melatonin was dissolved in ethanol having a 250 mM stock concentration before diluted with *α*-MEM. Melatonin at 1 or 100 *μ*M were incorporated into the culture medium after H_2_O_2_ treatment. Cells in the control (CTRL) group were treated with an equal volume of the vehicle (0.4 *μ*L ethanol/mL medium). BMMSCs were preincubated with 10 *μ*M of compound C (CC, Sigma-Aldrich) for 2 h to inhibit AMPK phosphorylation. Melatonin receptors were blocked by treating BMMSCs with 10 *μ*M of luzindole (an MT1/MT2 receptor antagonist) or 10 *μ*M of 4-phenyl-2-propionamidotetralin (4-P-PDOT; a selective MT2 receptor antagonist). Moreover, SIRT1 was inhibited through treating BMMSCs with 40 *μ*M of sirtinol (Sigma-Aldrich).

### 2.4. Cell Viability Assay

Cell viability was evaluated using a Cell Counting Kit-8 assay (CCK-8; Beyotime, Haimen, China). Briefly, BMMSCs were seeded into a 96-well plate, at a density of 1 × 10^4^ cells/well, and exposed to H_2_O_2_ for 2 h to induce cellular senescence. After 3 days, CCK-8 solution was added into each well and the cells incubated at 37°C for 1 h. Absorbance was determined at 450 nm using a PowerWave XS spectrophotometer (BioTek, Winooski, VT).

### 2.5. Senescence-Associated *β*-Galactosidase (SA-*β*-Gal) Staining

SA-*β*-gal activity in BMMSCs was assessed using a commercial kit (Beyotime) according to the manufacturer's instructions. Cells were fixed using 4% paraformaldehyde (Sigma-Aldrich) for 15 min, washed with phosphate-buffered saline (PBS) and incubated in *β*-gal solution overnight at 37°C without CO_2_. Nuclei were counterstained using 4′,6-diamidino-2-phenylindole (DAPI, Sigma-Aldrich), with *β*-gal-positive cells expected to stain blue. An inverted microscope (IX51, Olympus Corporation, Tokyo, Japan) was used to capture the digital images of SA-*β*-gal-positive cells. A total of at least 200 cells from 10 randomly chosen fields of each group were counted to calculate the ratio of senescent cells.

### 2.6. Analysis of Apoptosis

Detection of apoptosis was performed using an Apoptosis Kit (Thermo Fisher) according to the manufacturer's instructions. Briefly, BMMSCs were first detached using 0.25% trypsin-EDTA (Thermo Fisher Scientific) and then labeled with Alexa Fluor™ 488 dye-conjugated annexin V and propidium iodide (PI) for 15 min at room temperature. Cells were measured using a Cytomics FC500 Flow Cytometer (Beckman-Coulter, Brea, CA) and analyzed using Windows Multiple Document Interface for Flow Cytometry (WinMDI) 2.9 software.

### 2.7. Cell Cycle Analysis

BMMSCs were detached, fixed in 70% ethanol, and incubated at 4°C for 24 h. The fixed cells were then stained with 50 *μ*g/mL PI (Sigma-Aldrich), and RNA was digested using 50 *μ*g/mL RNase A (Sigma-Aldrich) with a 30 min incubation at 37°C in the dark. BMMSCs were measured using a Cytomics FC500 Flow Cytometer, and the data were analyzed using the MultiCycle AV DNA analysis software (Phoenix Flow Systems, San Diego, CA).

### 2.8. Osteogenic Induction and Evaluation of Matrix Mineralization

Osteogenic differentiation was induced in BMMSCs by culturing in osteogenic differentiation medium containing 10 mM *β*-glycerol phosphate, 100 nM dexamethasone, and 50 *μ*g/mL L-ascorbic acid (Sigma-Aldrich). After 14 days, cells were fixed in 4% paraformaldehyde for 30 min and incubated in 0.1% Alizarin Red S (ARS) solution (pH = 4.2, Sigma-Aldrich) at room temperature for 15 min. Digital images were captured using an inverted microscope (Olympus IX51). Matrix mineralization was quantified following the addition of 5% perchloric acid solution (Sigma-Aldrich) and subsequently determining the absorbance at 420 nm using a spectrophotometer (BioTek).

### 2.9. Quantitative Real-Time Polymerase Chain Reaction (qRT-PCR)

Total RNA was extracted using the TRIzol® reagent (Thermo Fisher Scientific) and reversely transcribed to complementary DNA (cDNA) by the RevertAid First-Strand cDNA Synthesis Kit (Thermo Fisher Scientific). Quantitative real-time reverse transcription-polymerase chain reaction (qRT-PCR) was performed on a CFX96™ Real-Time PCR System (Bio-Rad) using the iTap™ Universal SYBR® Green Supermix kit (Bio-Rad, Hercules, CA) according to the manufacturer's protocol. Relative transcript levels of target genes were calculated using the comparative Ct (2^−*ΔΔ*Ct^) method and expressed as a fold change respective to the control. The primer sequences are listed in Table 1.

### 2.10. Western Blot Analysis

Cells were first lysed in lysis buffer (Beyotime), containing protease inhibitors, for 1 h on ice, and total protein concentrations were determined using the BCA Protein Assay Kit (Beyotime). Equal amounts of cell lysates were then separated on a 10% polyacrylamide gel (Beyotime) and transferred to a nitrocellulose membrane (Beyotime). The membranes were blocked using a blocking buffer (Beyotime) for 30 min at room temperature and incubated with primary antibodies overnight at 4°C. The primary antibodies against P16 (1 : 5,000, ab108349), P21 (1 : 2,000, ab188224), P53 (1 : 2,000, ab1431), SIRT1 (1 : 2,000, ab110304), adenosine 5′-monophosphate-activated protein kinase (AMPK, 1 : 10,000, ab32047), phosphorylated AMPK (p-AMPK, 1 : 5,000, ab133448), RUNX2 (1 : 2,000, ab76956), SP7 (1 : 2,000, ab22552), BGLAP (1 : 2,000, ab13420), MT1 (1 : 1,000, ab203038), MT2 (1 : 1,000, ab203346), and *α*-tubulin (1 : 10,000, ab108349) were purchased from Abcam (Cambridge, UK). The membranes were then incubated with goat horseradish peroxidase-conjugated secondary anti-mouse antibody (1 : 5,000, ab6789, Abcam) or anti-rabbit antibody (1 : 10,000, ab6721, Abcam) for 1 h at room temperature, followed by visualization of bands using SuperSignal West Pico Substrate (Thermo Fisher Scientific) and X-OMAT BT Film (Beyotime). Gray values of the bands in scanned images were measured using ImageJ software (National Institutes of Health, Bethesda, MD), then normalized to that of *α*-tubulin before comparison.

### 2.11. Administration of Melatonin and Sirtinol *In Vivo*

OVX rats were injected with melatonin at 1 mg/kg (OVX+MT(L)) or 10 mg/kg (OVX+MT(H)) [23] via the tail vein for 3 months (twice per week). As controls, the sham and OVX groups were received saline containing the same amount of ethanol. The time for melatonin injection was between 10:00 and 10:30 AM daily with an infusion of about 5 seconds per rat to avoid possible physiological interference. Ovariectomized or sham-op rats were subjected to melatonin in combination with sirtinol at 1 mg/kg to inhibit SIRT1 [24].

At each predefined time point, 1 mL of blood from the left ventricle of melatonin-treated rats was collected and centrifuged. The supernatant was immediately frozen at -80°C for further analysis. Subsequently, serum concentrations of melatonin were determined using a commercial ELISA kit (E-EL-R0031c, Elabscience Biotechnology, Wuhan, China) according to the manufacturer's instructions [25].

### 2.12. Microcomputed Tomography (*μ*CT) Analysis

The *μ*CT analysis was performed using a Skyscan-1176 scanning system (Kontich, Belgium) as previously described [26]. Three-dimensional (3D) images were reconstructed and bone parameters were then calculated using the NRecon v1.6 and CTAn v1.13.8.1 software. BMD, bone volume ratio (BV/TV, %), trabecular number (Tb.N, mm^−1^), trabecular thickness (Tb.Th, mm), trabecular separation (Tb.Sp, mm), and bone surface/volume ratio (BS/BV, mm^−1^) in the secondary spongiosa region were evaluated.

### 2.13. Histological Analysis

Femur specimens were fixed with 4% paraformaldehyde and decalcified in 10% ethylene diaminetetraacetic acid (EDTA) for six weeks. Bone samples were subsequently dehydrated in graded EtOH solutions, embedded in paraffin, and sectioned into 5 *μ*m thick sections using a microtome. The paraffin sections were stained with hematoxylin and eosin (H&E) as previously described [26], and images captured using a bright field microscope (Zeiss Axiovert 200, Oberkochen, Germany).

### 2.14. Statistical Analysis

Data were presented as means ± standard errors of means (S.E.M.). One-way analysis of variance (ANOVA) was performed for multiple group comparison following the Tukey's post hoc test. Besides, independent two-tailed Student's *t*-test was used for two-group comparisons. Statistical analyses were performed using SPSS 13.0 statistical software (SPSS Inc., Chicago, IL) where ^∗^*p* < 0.05 and ^∗∗^*p* < 0.01 were considered significant.

## 3. Results

### 3.1. OVX BMMSCs Exhibited Reduced Resistance to H_2_O_2_-Induced Cellular Senescence

**Figure 1:**
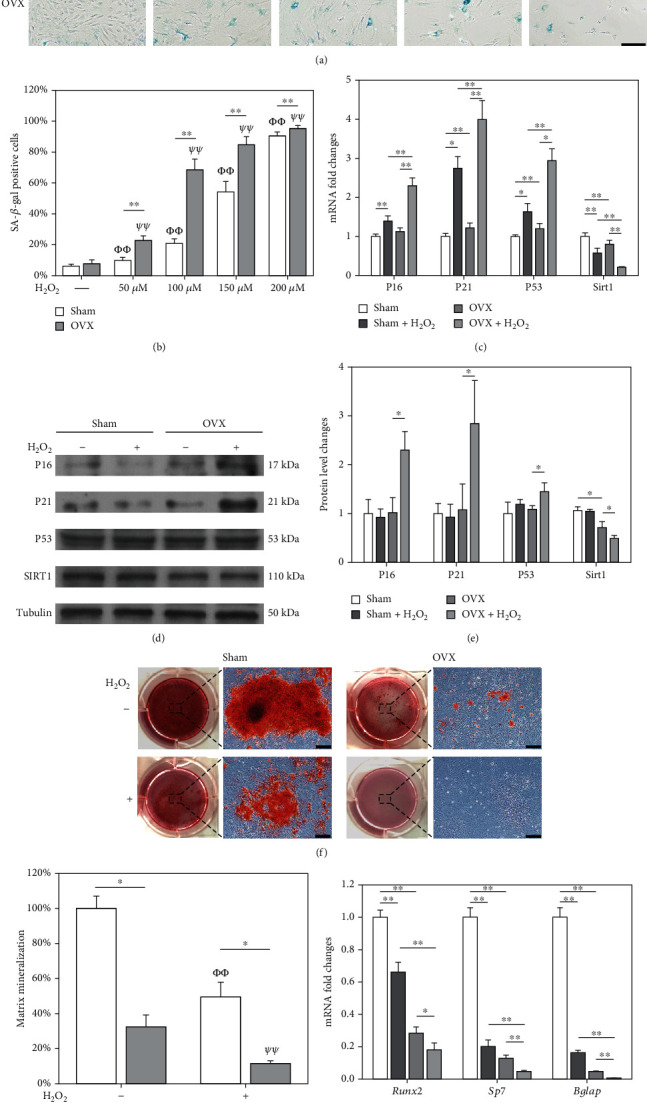
Evaluation of the resistance of BMMSCs to oxidative stress-induced premature senescence. BMMSCs from sham-operated (sham) and ovariectomized (OVX) rats were exposed to hydrogen peroxide (H_2_O_2_) ranging from 50 *μ*M to 200 *μ*M for 2 h and cultured for an additional 72 h. (a, b) Senescence-associated *β*-galactosidase (SA-*β*-gal) staining was performed to label senescent cells. Scale bar = 100 *μ*m. (c) The mRNA expression levels of *P16*, *P21*, *P53*, and *Sirt1* were quantified using qRT-PCR. (e) The protein levels of P16, P21, P53, and SIRT1 were determined using Western blot assays. (f) After exposure to 100 *μ*M of H_2_O_2_, BMMSCs were induced toward the osteogenic differentiation for 14 days. Representative images of mineralized extracellular matrix stained by Alizarin Red S (ARS). Scale bar = 200 *μ*m. (g) Quantification of the stained mineral layers in H_2_O_2_-treated and untreated BMMSCs. The values shown were normalized to those of the untreated sham BMMSCs. (h) The mRNA levels of osteoblast-specific marker genes, including *Runx2*, *Sp7*, and *Bglap*, were quantified with qRT-PCR using *Gapdh* as the reference gene. Data are shown as the mean ± S.E.M of six independent experiments (*n* = 6) in SA-*β*-gal staining, four independent experiments (*n* = 4) in ARS assays, four independent experiments (*n* = 4) in qRT-PCR experiments, and three independent experiments (*n* = 3) in Western blot assays. Statistically significant differences are indicated by ^∗^*p* < 0.05 or ^∗∗^*p* < 0.01 between the indicated groups; *^Φ^p* < 0.05 or *^ΦΦ^p* < 0.01 versus untreated sham BMMSCs; *^Ψ^p* < 0.05 or *^ΨΨ^p* < 0.01 versus untreated OVX BMMSCs.

SA-*β*-gal staining (Figures 1(a) and 1(b)) and cell viability assays (Supplementary Fig. 1A) showed that H_2_O_2_ induced senescence in a dose-dependent manner. Notably, on H_2_O_2_ exposure with the same concentrations (e.g., 68.5 ± 6.9% vs. 20.9 ± 2.8% at 100 *μ*M), the percentage of *β*-gal-positive cells in the OVX group were markedly higher than the sham group. Since the sublethal dosage of H_2_O_2_ at 100 *μ*M had no serious apoptosis (Supplementary Fig. 1B&C), it was selected for subsequent experiments. Cell cycle distribution indicated a G0/G1 cell cycle arrest in OVX BMMSCs due to the high proportion in the G0/G1 phase but low percentages in S phase (Supplementary Fig 1D&E). Real-time PCR showed that exposure to H_2_O_2_ significantly upregulated the transcript levels of *P16*, *P21*, and *P53* by 1.0-fold, 2.3-fold, and 1.5-fold in OVX BMMSCs, respectively, while the mRNA level of *Sirt1* was decreased by 73.1% (Figure 1(c)). Western blot assays confirmed that the protein expression of P16, P21, and P53 in the OVX+H_2_O_2_ group was significantly increased, while SIRT1 expression was downregulated (Figures 1(d) and 1(e)). After a 14-day osteogenic induction, senescent sham and OVX BMMSCs showed decreased levels of both matrix mineralization (Figures 1(f) and 1(g)) and osteoblast-specific marker genes (Figure 1(h)).

### 3.2. Protection of OVX BMMSCs against Cellular Senescence by In Vitro Treatments with Melatonin

**Figure 2:**
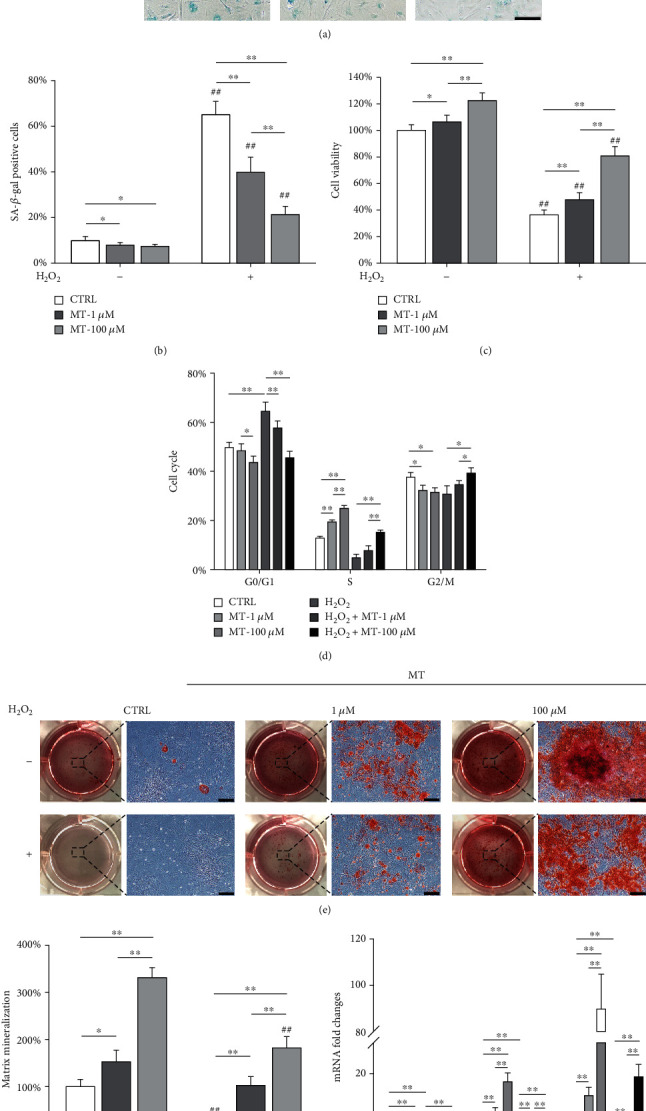
Melatonin treatments protected OVX BMMSCs from H_2_O_2_-induced premature senescence. OVX BMMSCs were first exposed to H_2_O_2_ (100 *μ*M) for 2 h and then treated with melatonin (MT) at 1 *μ*M and 100 *μ*M for an additional 72 h. (a, b) Senescent cells were labeled with senescence-associated *β*-galactosidase (SA-*β*-gal) staining. Scale bar = 100 *μ*m. (c) The cell viability of OVX BMMSCs was determined by CCK-8 assays. (d) Analysis of cell cycle distribution showed that melatonin treatments prevented cell cycle arrest in OVX BMMSCs. (e) Melatonin rescued the osteogenic differentiation of H_2_O_2_-treated OVX BMMSCs. After a 14-day osteogenic induction, the mineralized extracellular matrix was stained by Alizarin Red S (ARS). Scale bar = 200 *μ*m. (f) Quantification of the stained mineral layers in H_2_O_2_- and melatonin-treated cells. The values were normalized to those of the untreated OVX BMMSCs. (g) The mRNA levels of osteoblast-specific marker genes, including *Runx2*, *Sp7*, and *Bglap*, were quantified with qRT-PCR using *Gapdh* as the reference gene for normalization. Values are presented as the mean ± S.E.M of six independent experiments (*n* = 6) in SA-*β*-gal staining, eight independent experiments (*n* = 8) in cell viability assays, three independent experiments (*n* = 3) in cell cycle assays, four independent experiments (*n* = 4) in ARS assays, and four independent experiments (*n* = 4) in qRT-PCR experiments. Statistically significant differences are indicated by ^∗^*p* < 0.05 or ^∗∗^*p* < 0.01 between the indicated groups. Statistically significant differences are indicated by ^∗^*p* < 0.05 or ^∗∗^*p* < 0.01 between the indicated groups; ^#^*p* < 0.05 or ^##^*p* < 0.01 versus the CTRL group.

Following the H_2_O_2_ exposure (100 *μ*M), OVX BMMSCs were treated with 1 or 100 *μ*M of melatonin. Melatonin-treated cells showed decreased *β*-gal-positive cells (39.8 ± 6.6% and 21.3 ± 3.5%, respectively) than the CTRL group (65.0 ± 5.8%) (Figures 2(a) and 2(b)). Consistently, melatonin improved the cell viability by 31.3% at 1 *μ*M and 122.2% at 100 *μ*M (Figure 2(c)). Analysis of cell cycle distribution confirmed that melatonin increased the proportion of cells in S phase while decreasing the proportion of cells in G0/G1 phase (Figure 2(d), Supplementary Fig. 2A). More importantly, melatonin restored the osteogenic capacity of senescent OVX BMMSCs by promoting mineral deposition (2.4-fold at 1 *μ*M and 5.1-fold at 100 *μ*M; Figures 2(e) and 2(f)) and upregulating the osteoblast-specific markers at both the transcript (Figure 2(g)) and protein levels (Supplementary Fig. 2B&C).

We also investigated the effect of melatonin on BMMSCs from sham-op rats. Exposure to 100 *μ*M of H_2_O_2_ resulted in 17.7% ± 2.4%*β*-gal-positive cells, whereas treatment with melatonin of 1 and 100 *μ*M reduced the ratio of senescent cells to 11.1% ± 1.9% and 7.1% ± 1.2%, respectively (Supplementary Fig. 3A&B). The cell viability of H_2_O_2_-treated cells was increased by 100 *μ*M of melatonin (Supplementary Fig. 3C), and the gene expression levels of senescence markers such as *P16*, *P21*, and *P53* were significantly downregulated (Supplementary Fig. 3D). Meanwhile, treatment with melatonin improved the osteogenic differentiation of H_2_O_2_-treated BMMSCs, as evidenced by the enhanced matrix mineralization (Supplementary Fig. 4A&B) and gene expression levels of osteoblast-specific markers (Supplementary Fig. 4C).

### 3.3. Melatonin Activated the AMPK-SIRT1 Signaling Pathway in Senescent OVX BMMSCs

**Figure 3:**
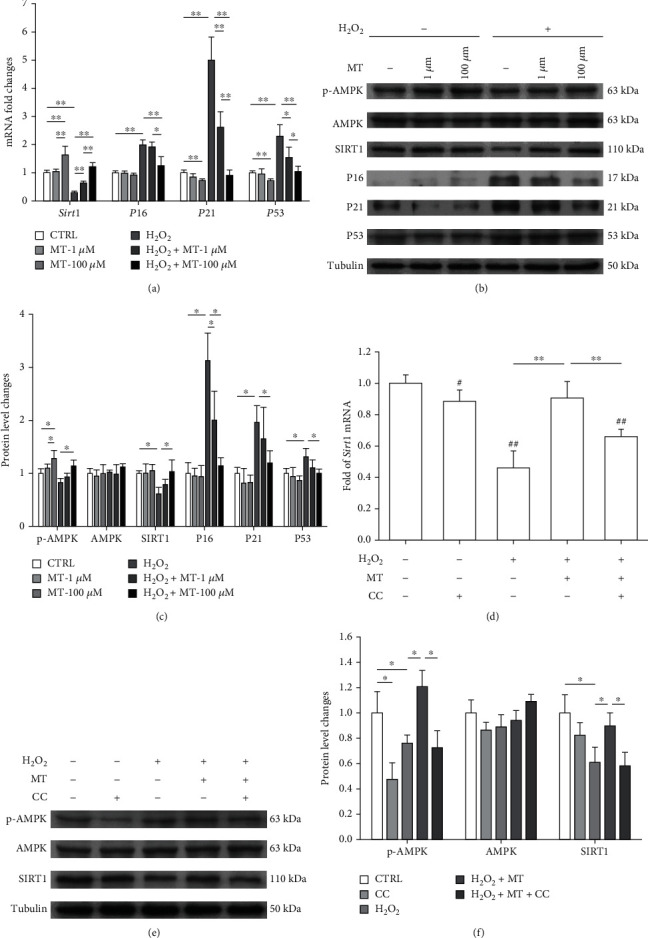
Melatonin prevented H_2_O_2_-induced premature senescence through the AMPK-SIRT1 signaling pathway. OVX BMMSCs were first exposed to H_2_O_2_ (100 *μ*M) for 2 h and then treated with melatonin (MT) at 1 *μ*M or 100 *μ*M for an additional 72 h. (a) The mRNA expression levels of *Sirt1*, *P16*, *P21*, and *P53* were quantified. (b, c) The protein levels of p-AMPK, AMPK, SIRT1, P16, P21, and P53 were determined using Western blot assays. (d) To inhibit the phosphorylation of AMPK, OVX BMMSCs were pretreated with 10 *μ*M of compound C (CC) and then treated with 100 *μ*M of melatonin. The mRNA expression levels of *Sirt1* were quantified using qRT-PCR. (e, f) The protein levels of p-AMPK, AMPK, and SIRT1 were determined using Western blot assays. Values are presented as the mean ± S.E.M of four independent experiments (*n* = 4) in qRT-PCR experiments and three independent experiments (*n* = 3) in Western blot assays. Statistically significant differences are indicated by ^∗^*p* < 0.05 or ^∗∗^*p* < 0.01 between the indicated groups.

We next investigated the underlying mechanisms of melatonin-mediated protection of OVX BMMSCs from oxidative stress. Melatonin at 100 *μ*M upregulated the mRNA expression of *Sirt1* by 315.4% and reduced *P16* and *P21* and by *P53* by 37.2%, 81.8%, and 54.6%, respectively, in H_2_O_2_-treated OVX BMMSCs (Figure 3(a)). Western blot results showed that melatonin treatment significantly increased the phosphorylation of AMPK and SIRT1 protein expressions (Figures 3(b) and 3(c)). The protein levels of P16, P21, and P53 in H_2_O_2_-treated OVX BMMSCs were significantly downregulated by melatonin treatments, suggesting that melatonin prevents stem cell senescence through upregulation of SIRT1. To explore the AMPK pathway in melatonin-mediated antisenescence effect, OVX BMMSCs were treated with CC to inhibit p-AMPK before melatonin treatment. Inhibition of AMPK phosphorylation significantly decreased the mRNA level of *Sirt1* by 27.2% (Figure 3(d)) and the protein level by 35.2% (Figures 3(e) and 3(f)).

**Figure 4:**
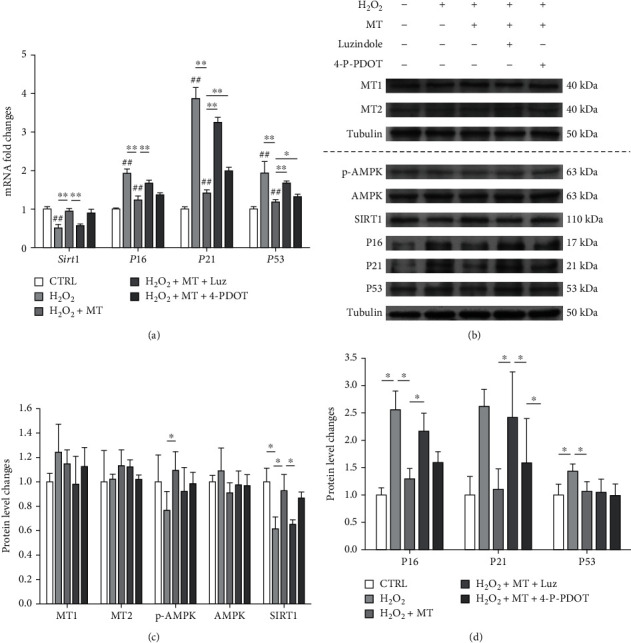
Melatonin-mediated antisenescence effect was via MT1 and MT2 membrane receptors. To investigate the role of melatonin receptors, OVX BMMSCs were pretreated with 10 *μ*M of luzindole and 10 *μ*M of 4-phenyl-2-propionamidotetralin (4-P-PDOT), respectively, and then treated with H_2_O_2_ (100 *μ*M) for 2 h and melatonin (MT, 100 *μ*M) for an additional 72 h. (a) The mRNA expression levels of *Sirt1*, *P16*, *P21*, and *P53* were quantified using real-time qRT-PCR. (b–d) The protein levels of MT1, MT2, p-AMPK, AMPK, SIRT1, P16, P21, and P53 were determined using Western blot assays. Values are presented as the mean ± S.E.M of four independent experiments (*n* = 4) in qRT-PCR experiments and three independent experiments (*n* = 3) in Western blot assays. Statistically significant differences are indicated by ^∗^*p* < 0.05 or ^∗∗^*p* < 0.01 between the indicated groups; ^#^*p* < 0.05 or ^##^*p* < 0.01 versus the control (CTRL) group.

To investigate the involvement of melatonin receptors, two inhibitors luzindole and 4-P-PDOT were used before melatonin treatment. Consequently, the addition of luzindole downregulated the gene expression of *Sirt1* 39.8%, whereas *P21* was upregulated by 129.7% and 40.6% following the addition of both luzindole and 4-P-PDOT, respectively (Figure 4(a)). Western blot assays confirmed that either luzindole or 4-P-PDOT impeded the protective effect of melatonin, but the protein levels of MT1 and MT2 were not affected by melatonin receptor inhibitors (Figures 4(b)–4(d)). These results suggested that melatonin activated the AMPK-SIRT1 signaling pathway via melatonin receptors.

### 3.4. *In Vivo* Administration of Melatonin Ameliorated Estrogen Deficiency-Induced Bone Loss by Preserving the Antisenescence Functions of BMMSCs

**Figure 5:**
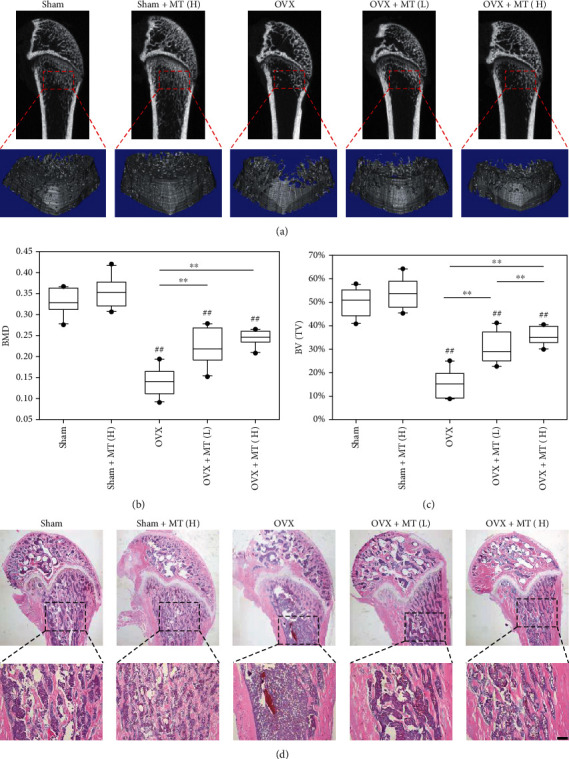
*In vivo* administration of melatonin protected the trabecular bone microstructure of OVX rats following estrogen withdrawal. After ovariectomy, melatonin (MT) was injected into OVX rats through the tail vein at a low dosage (1 mg/kg, OVX+MT(L)) or a high dosage (10 mg/kg, OVX+MT(H)), while sham-op rats were injected with melatonin at 10 mg/kg (sham+MT(H)). The sham and OVX rats received saline containing the same amount of ethanol. (a) Micro-CT and 3D reconstruction were used to histomorphometrically analyze the rat femurs. (b) The effect of melatonin administration on bone mineral density (BMD). (c) The effect of melatonin administration on bone volume ratio (BV/TV, %). (d) Representative histological images of rat femurs stained by hematoxylin and eosin (H&E). Scale bar = 200 *μ*m. Values are presented as the mean ± S.E.M of ten samples in each group (*n* = 10) in micro-CT and 3D reconstruction assays. Statistically significant differences are indicated by ^∗^*p* < 0.05 or ^∗∗^*p* < 0.01 between the indicated groups; ^#^*p* < 0.05 or ^##^*p* < 0.01 versus the sham group.

After intravenous injection with melatonin (1 and 10 mg/kg), serum concentrations of melatonin in OVX rats versus time were shown in Supplementary Fig. 5A. Melatonin administration successfully prevented bone deterioration in OVX rats (Figure 5(a)). Three-dimensional reconstruction indicated that BMD and BV/TV in the OVX+MT (H) group was increased by 129.7% and 74.7%, respectively (Figures 5(b) and 5(c)). Melatonin administration also improved the values of BS/TV and Tb.N while simultaneously reducing Tb.Sp and BS/BV in OVX rats (Supplementary Fig. 5B-E). The protective effect of melatonin on the bone micro-structure was determined by histological experiments (Figure 5(d)).

**Figure 6:**
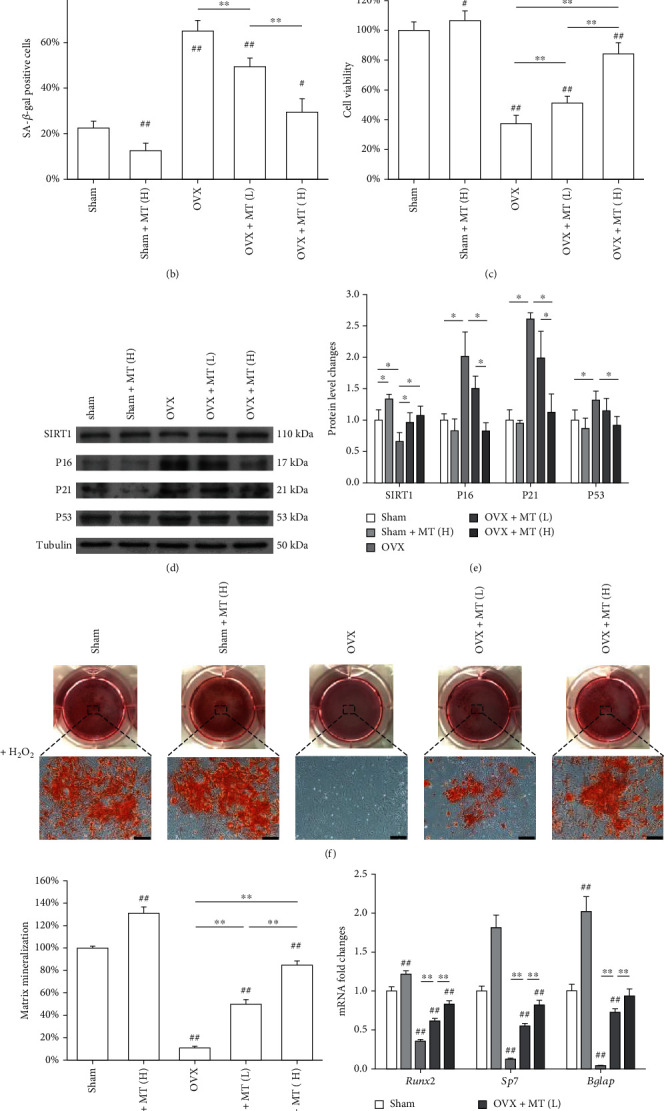
Evaluation of the antisenescence properties of BMMSCs derived from melatonin-treated rats. BMMSCs were isolated from untreated and melatonin-treated (MT) sham-op or OVX rats and then exposed to H_2_O_2_ (100 *μ*M) for 2 h. (a, b) Senescent cells were labeled by senescence-associated *β*-galactosidase (SA-*β*-gal) staining. Scale bar = 100 *μ*m. (c) The cell viability of OVX BMMSCs was determined by CCK-8 assays. (d, e) The protein levels of SIRT1, P16, P21, and P53 were determined using Western blot assays. (f) After exposure to H_2_O_2_, BMMSCs were induced toward osteogenic differentiation. Matrix mineralization was determined by Alizarin Red S (ARS) staining. Scale bar = 200 *μ*m. (g) The stained mineral layers were quantified. The values were normalized to those of the sham group. (h) The mRNA levels of osteoblast-specific marker genes, including *Runx2*, *Sp7*, and *Bglap* were quantified using qRT-PCR with *Gapdh* serving as the internal reference gene for normalization. Values are presented as the mean ± S.E.M of six independent experiments (*n* = 6) in SA-*β*-gal staining, eight independent experiments (*n* = 8) in cell viability assays, four independent experiments (*n* = 4) in ARS assays, three independent experiments (*n* = 3) in Western blot assays, and four independent experiments (*n* = 4) in qRT-PCR experiments. Statistically significant differences are indicated by ^∗^*p* < 0.05 or ^∗∗^*p* < 0.01 between the indicated groups; ^#^*p* < 0.05 or ^##^*p* < 0.01 versus the sham group.

Furthermore, BMMSCs were isolated from melatonin-treated OVX rats and exposed to H_2_O_2_. SA-*β*-gal-positive cells were significantly lower in both the OVX+MT(L) (49.4 ± 3.8%) and OVX+MT(H) (25.9 ± 5.9%) groups than the OVX (65.0% ± 4.6%) group (Figures 6(a) and 6(b)). Besides, the cell viability was significantly higher in the melatonin-treated groups (Figure 6(c)). The transcript level of *Sirt1* was 359.9% higher in the OVX+MT(H) group, while *P16* was 36.6% and *P21* was 46.5% lower than the OVX group (Supplementary Fig. 6). Western blot assays revealed that melatonin administration upregulated SIRT1 but downregulated P16, P21, and P53 at their protein levels (Figures 6(d) and 6(e)). BMMSCs derived from melatonin-treated OVX rats showed higher levels of matrix mineralization and osteoblast-specific marker gene expression even after subjected to H_2_O_2_ (Figures 6(f)–6(h)).

### 3.5. Inhibition of SIRT1 Abolished the Antisenescence Effects of Melatonin

**Figure 7:**
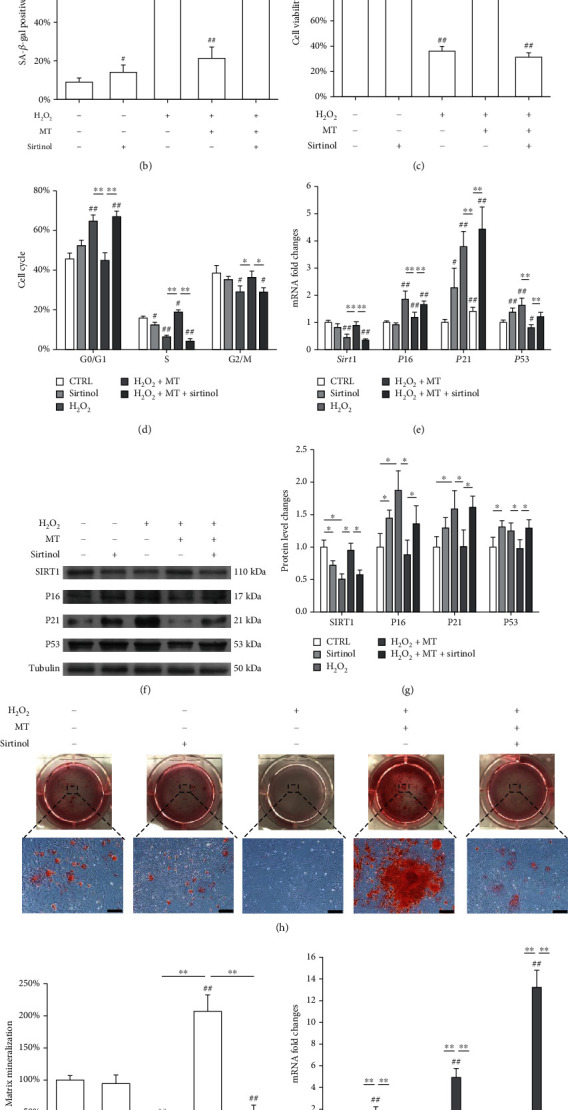
Inhibition of SIRT1 by sirtinol counteracted the antisenescence effects of melatonin on OVX BMMSCs. *In vitro* cultured OVX BMMSCs were first exposed to H_2_O_2_ for 2 h and then treated with 100 *μ*M of melatonin (MT) with or without sirtinol (40 *μ*M) for 72 h. (a, b) Senescent cells were labeled by senescence-associated *β*-galactosidase (SA-*β*-gal) staining. Scale bar = 100 *μ*m. (c) The effect of sirtinol treatment on cell viability was determined by CCK-8 assays. (d) The effect of sirtinol treatment on cell cycle distribution was evaluated by flow cytometry. (e) The mRNA expression levels of *Sirt1*, *P16*, *P21*, and *P53* were quantified using qRT-PCR. (f, g) The protein levels of SIRT1, P16, P21, and P53 were determined using Western blot assays. (h) Sirtinol-treated BMMSCs were induced toward osteogenic differentiation. Matrix mineralization was determined by Alizarin Red S (ARS) staining. Scale bar = 200 *μ*m. (i) The stained mineral layers were quantified. The values were normalized to those of the sham group. (j) The mRNA levels of osteoblast-specific marker genes, including *Runx2*, *Sp7*, and *Bglap*, were quantified with qRT-PCR in which *Gapdh* was used for normalization. Values are presented as the mean ± S.E.M of six independent experiments (*n* = 6) in SA-*β*-gal staining, eight independent experiments (*n* = 8) in cell viability assays, three independent experiments (*n* = 3) in cell cycle assays, four independent experiments (*n* = 4) in ARS assays, and four independent experiments (*n* = 4) in qRT-PCR experiments, and three independent experiments (*n* = 3) in Western blot assays. Statistically significant differences are indicated by ^∗^*p* < 0.05 or ^∗∗^*p* < 0.01 between the indicated groups; ^#^*p* < 0.05 or ^##^*p* < 0.01 versus the sham group.

To explore the underlying mechanism involved the SIRT1 signaling pathway, *in vitro* cultured OVX BMMSCs were treated with a SIRT1 inhibitor sirtinol (40 *μ*M) and melatonin (100 *μ*M). Treatment with sirtinol completely counteracted the protective effect of melatonin on stem cell senescence due to the high percentage of SA-*β*-gal-positive cells (62.6 ± 5.4%; Figures 7(a) and 7(b)) with a noticeable decrease in cell viability (Figure 7(c)). Consistently, sirtinol reduced the proportion of cells in the S phase to 4.2 ± 1.3% (Figure 7(d), Supplementary Fig. 7). Sirtinol upregulated *P16*, *P21*, and *P53* by 40.0%, 2.1%, and 50.8%, respectively, but downregulated *Sirt1* by 60.8% (Figure 7(e)). The effects of sirtinol on SIRT1 and cell cycle regulators were demonstrated through the Western blot assays (Figures 7(f) and 7(g)). When inducing toward osteogenesis, the levels of matrix mineralization and osteoblast-specific gene expression were suppressed by sirtinol treatment (Figures 7(h)–7(j)).

**Figure 8:**
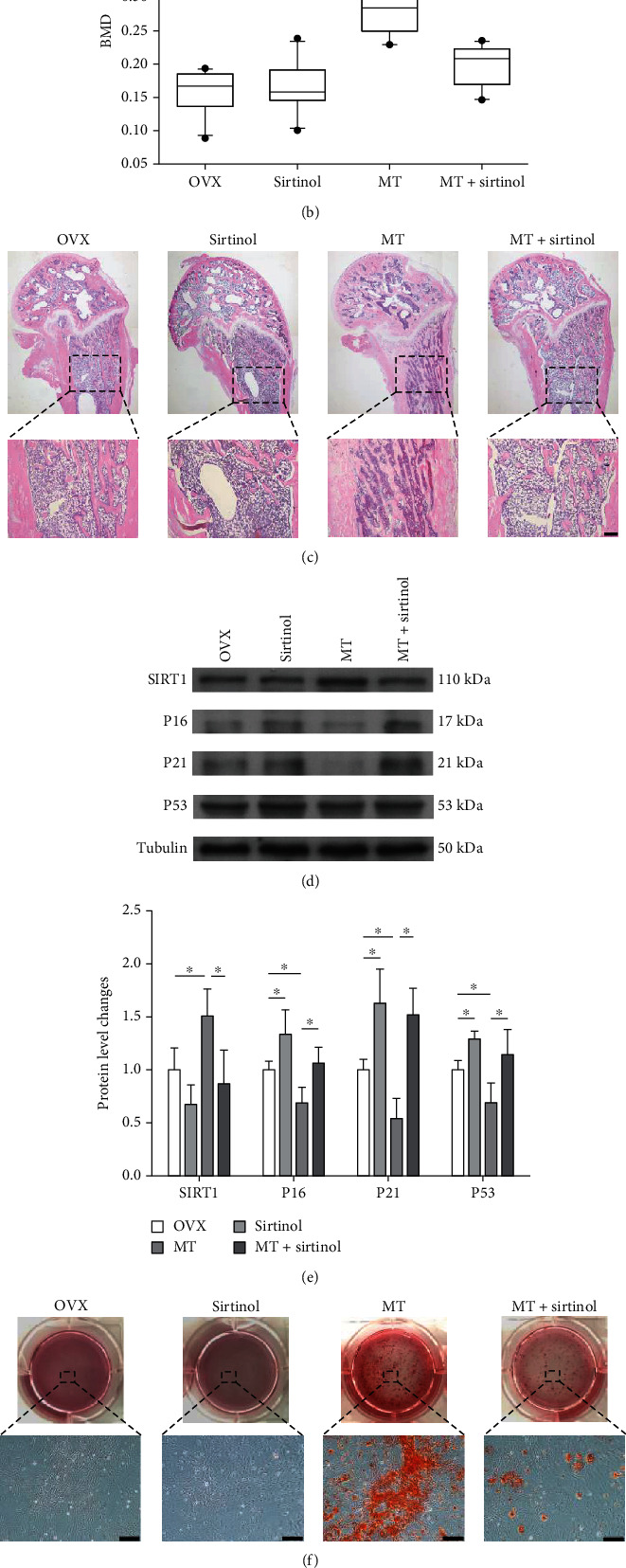
Injection of sirtinol aggravated bone loss in melatonin-treated OVX rats. OVX rats were injected with melatonin (MT, 10 mg/kg) or sirtinol (1 mg/kg) through the tail vein. (a) Micro-CT and 3D reconstruction were used to analyze the trabecular bone microstructure. (b) The effect of sirtinol treatment on bone mineral density (BMD). (c) Representative histological images of rat femurs stained by hematoxylin and eosin (H&E). Scale bar = 200 *μ*m. (d, e) BMMSCs were isolated from melatonin- and sirtinol-treated OVX rats, exposed to H_2_O_2_ (100 *μ*M) for 2 h, and cultured for an additional 72 h. The protein levels of SIRT1, P16, P21, and P53 were determined using Western blot assays. (f) BMMSCs derived from melatonin- or sirtinol-treated OVX rats were induced toward osteogenic differentiation. Matrix mineralization was assessed by Alizarin Red S (ARS) staining. Scale bar =200 *μ*m. (G) The stained mineral layers were quantified. The values shown were normalized to those of the sham group. Values are presented as the mean ± S.E.M of ten samples in each group (*n* = 10) in micro-CT and 3D reconstruction assays, four independent experiments (*n* = 4) in ARS assays, and three independent experiments (*n* = 3) in Western blot assays. Statistically significant differences are indicated by ^∗^*p* < 0.05 or ^∗∗^*p* < 0.01 between the indicated groups; ^#^*p* < 0.05 or ^##^*p* < 0.01 versus the sham group.

To further investigate the role of SIRT1, OVX or sham rats were injected with sirtinol and melatonin. The *μ*CT results showed that the trabecular bone microstructure of melatonin-treated OVX rats was deteriorated following the sirtinol injection (Figure 8(a)) with 41.3% decrease in BMD (Figure 8(b)). However, sirtinol treatment consistently reduced BV/TV, BS/TV, and Tb.N, while increasing Tb.Sp and BS/BV in melatonin-treated OVX rats (Supplementary Fig. 8A-E). The results of H&E staining proved that sirtinol treatment terminated the protective effect of melatonin on bone microstructure (Figure 8(c)). Furthermore, the transcript level of *Sirt1* was reduced by 54.8% in the MT+sirtinol group, while *P16*, *P21*, and *P53* were significantly upregulated by 42.9%, 59.5%, and 24.4%, respectively (Supplementary Fig. 8F). The protein levels of SIRT1, P16, P21, and P53 were consistent with their gene expression (Figures 8(d) and 8(e)). The calcium deposition was decreased by 66.1% (Figures 8(f) and 8(g)), and the expression of osteoblast-specific markers was also significantly downregulated following the sirtinol treatment (Supplementary Fig. 8G). In addition, we examine the effect of sirtinol treatment on sham-op rats. As shown in Supplementary Fig. 9A&B, sirtinol significantly decreased BMD of sham-op rats with or without melatonin treatment. The evaluation of BMMSCs derived from sirtinol-treated sham rats demonstrated that sirtinol downregulated the expression of SIRT1, while increasing the expression of P16 and P21 (Supplementary Fig. 9C-E). Meanwhile, the cells showed attenuated osteogenic differentiation, as evidenced by the weak matrix mineralization and low expression of osteoblast-specific markers (Supplementary Fig. 10).

## 4. Discussion

Previous studies have established that the biological properties of BMMSCs are altered in OP patients [22]. Consistent with other studies, a significant decrease in the osteogenic capacity of OVX rat-derived BMMSCs was evident [27]. Based on studies, none showed estrogen deficiency directly induced stem cell senescence. On the contrary, a previous study has indicated that some senescence characteristics were detectable in OVX BMMSCs [8]. Briefly, exposure to a low level of oxidative stress rapidly induced senescence in OVX BMMSCs. Thus, to our knowledge, this is the first report to prove that estrogen deficiency results in a weak resistance of stem cells to oxidative stress-induced cellular senescence. Functionally, stress-induced senescence aggravates the impairment in BMMSC osteogenic capacity, leading to suppressed matrix synthesis and poor bone formation in OP patients.

From previous study findings, elimination of senescent cells in old mice not only improved bone formation but also suppressed bone resorption [7]. During the long-term expansion, melatonin supplementation preserved the functional properties of stem cells and enhanced their therapeutic functions by inhibiting senescence phenotypes [28]. In response to some pathological stimuli such as iron overload [29] and uremic toxin exposure [30], melatonin effectively protects MSC differentiation potential by preventing premature senescence. In this study, *in vitro* treatments with melatonin successfully improved resistance of OVX BMMSCs to senescence. Furthermore, intravenous administration of melatonin significantly preserved their antisenescence properties, suggesting that melatonin-based therapy could be a promising strategy for treating postmenopausal OP patients.

Increased expression of P21 was reported in late-passaged MSCs whereas knockdown of *P21* by shRNAs rescued their capacity for bone repair [31]. In this study, P21 was inhibited by melatonin in senescent BMMSCs, possibly through deacetylation of P53. The study of Han et al. showed that melatonin improved the functional survival of adipose-derived MSCs in infarcted hearts by decreasing Ac-P53 expression [32]. We observed that the P16 expression in senescent BMMSCs, another main regulator of cellular senescence [33], was also suppressed by melatonin. The nicotinamide phosphoribosyltransferase- (NAMPT-) SIRT1 axis may be involved, both of which can be upregulated by melatonin [34] and overexpression of NAMPT was able to ameliorate senescence-associated phenotypic features in late-passaged MSCs [35]. However, further studies are needed to understand the different roles of melatonin in regulating P21 and P16 during stem cell senescence.

SIRT1, an important target for energy metabolism, has been found to be involved in melatonin-mediated antisenescence effects. Activation of SIRT1 potentially improves the self-renewal capacity of BMMSCs by protecting sex-determining region Y- (SRY-) box 2 (SOX2) from ubiquitination [36]. *In vivo* experiments confirmed that overexpression of SIRT1 in MSCs protected against bone loss in mice by enhancing the transcriptional activity of class O subfamily of forkhead box 3A (FOXO3A) [37]. Age-related senescence phenotypes in MSCs can also be attenuated by SIRT1 overexpression to improve telomerase activity and prevent DNA damage [38]. Therefore, our findings confirmed that melatonin prevents cellular senescence in OVX BMMSCs through SIRT1, because inhibition of SIRT1 by sirtinol terminated the protective effects of melatonin on H_2_O_2_-induced senescence. These results were consistent with a previous study that melatonin alleviated doxorubicin-induced acute cardiac dysfunction in mice through activation of AMPK, while blockade of AMPK compromised the cardio-protective action of melatonin [39].

To investigate the roles of MT1 and MT2 receptors in melatonin-mediated antisenescence, we treated OVX BMMSCs with either luzindole or 4-P-PDOT. Interestingly, both inhibitors counteracted the protective effects of melatonin, though 4-P-PDOT showed more potent effect than luzindole. According to previous studies, melatonin promoted bone formation through MT2 receptors and knockout of MT2 in mice resulting in a significant low bone mass rather than MT1 receptors [15]. However, the consistency in this study was possibly caused by estrogen deficiency that might affect the biological functions of melatonin through different receptors. Melatonin was shown to inhibit cell proliferation of human breast cancer cells by binding to MT1 receptors and suppressing estrogen-induced estrogen receptor alpha (ER*α*) transcriptional activity [40]. Estrogen-stimulated mammary gland development was repressed by elevated MT1 receptor expression, suggesting that melatonin could modulate the estrogen response pathway through MT1 receptors [41]. Hence, the specific roles of both MT1 and MT2 receptors in modulating the biological functions of melatonin in estrogen-deficient patients are still unclear and recommended on their further explorations.

In addition to estrogen deficiency-induced OP, melatonin with antisenescence effects may benefit senile OP subjects. Senescent cells accumulated in the bone microenvironment drive bone loss when ageing [42]. Dietary melatonin supplementation can effectively improve the microstructure and biomechanical properties of bones in aged rats [43]. A possible mechanism is that melatonin protects the structural and functional integrity of vascular endothelium against ageing-induced damage [44]. However, another potential mechanism may involve its antioxidant properties. In elderly primary essential hypertensive patients, administration of 5 mg/day of melatonin improved their antioxidant defense functions, as demonstrated by a significant increase in superoxide dismutase (SOD) 1 and catalase activities as well as a reduction in the serum malondialdehyde level [45]. In OVX rats, melatonin attenuated ROS levels by upregulating mitochondrial antioxidant enzymes (e.g., SOD2 and glutathione peroxidase 1) [26]. Thus, the potential of melatonin to protect aged BMMSCs from senescence by improving their antioxidant functions necessitates further studies.

## 5. Conclusions

**Figure 9:**
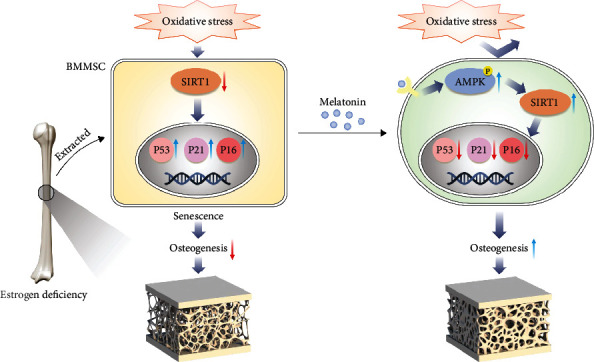
A schematic diagram illustrating the underlying mechanism of melatonin-mediated anti-senescence effect in OVX rats. Estrogen deficiency results in a bone loss and bone microstructure deterioration. BMMSCs derived from OVX rats (OVX BMMSCs) show that estrogen deficiency did not directly cause stem cell senescence, but exposure to low levels of oxidative stress rapidly induced premature senescence in OVX BMMSCs. Melatonin prevents oxidative stress-induced senescence in OVX BMMSCs and subsequently restores their impaired osteogenic capacity via activation of the AMPK-SIRT1 signaling pathway through melatonin receptors. Intravenous administration of melatonin ameliorates bone loss in OVX rats and preserves the anti-senescence property of BMMSCs.

We demonstrate that estrogen deficiency did not directly cause stem cell senescence, but exposure to low levels of oxidative stress rapidly induced premature senescence in OVX BMMSCs. As illustrated in Figure 9, melatonin prevented oxidative stress-induced senescence in OVX BMMSCs and subsequently restored their impaired osteogenic capacity via activation of the AMPK-SIRT1 signaling pathway through melatonin receptors. Intravenous administration of melatonin-ameliorated bone loss in OVX rats and preserved the antisenescence property of BMMSCs. Herein, melatonin treatment represents a novel strategy for managing postmenopausal OP patients by enhancing the resistance of BMMSCs to cellular senescence.

## Figures and Tables

**Table 1:** Primers used for quantitative real-time RT-PCR.

Gene	Forward primer sequence (5′-3′)	Reverse primer sequence(5′-3′)
*Gapdh*	GCAAGTTCAACGGCACAG	CGCCAGTAGACTCCACGAC
*P16*	ATGGAGTCCTCTGCAGATAGA	ATCGGGGTACGACCGAAAGTGTT
*P21*	AAGAGGCCCAGTACCTCCTC	GGCGCTTGGAGTGATAGAAA
*P53*	CTTCGAGATGTTCCGAGAGC	CTTCGGGTAGCTGGAGTGAG
*Sirt1*	GAAAATGCTGGCCTAATAGACTTG	TGGTACAAACAAGTATTGATTACCG
*Runx2*	CCAACTTCCTGTGCTCCGTG	GTGAAACTCTTGCCTCGTCCG
*Sp7*	CCCAACTGTCAGGAGCTAGAG	GATGTGGCGGCTGTGAAT
*Bglap*	GACCCTCTCTCTGCTCACTCT	GACCTTACTGCCCTCCTGCTTG

## Data Availability

The original contributions presented in the study are included in the article/supplementary material; further inquiries can be directed to the corresponding author.

## Supplementary Materials

Supplementary Materials Supplementary Figure 1: evaluation of oxidative stress-induced premature senescence and apoptosis of sham and OVX BMMSCs. Supplementary Figure 2: the effects of melatonin on the cell cycle distribution and osteogenic differentiation of in H_2_O_2_-treated OVX BMMSCs. Supplementary Figure 3: the effect of melatonin on sham BMMSCs in the presence of H_2_O_2_-induced premature senescence. Supplementary Figure 4: melatonin improved the osteogenic differentiation of H_2_O_2_-treated sham BMMSCs. Supplementary Figure 5: intravenous injection of melatonin protected the trabecular bone micro-structure of OVX rats. Supplementary Figure 6: evaluation of the gene expression pattern of BMMSCs derived from melatonin-treated rats. Supplementary Figure 7: inhibition of SIRT1 by sirtinol aggravated premature senescence in OVX BMMSCs. Supplementary Figure 8: the effects of sirtinol on the trabecular bone microstructure of melatonin-treated OVX rats. Supplementary Figure 9: sirtinol treatment attenuated the antisenescence effect of melatonin on sham BMMSCs. Supplementary Figure 10: sirtinol treatment suppressed the osteogenic effect of melatonin on sham BMMSCs.
