# Supplementary material for: Melatonin Improves the Resistance of Oxidative Stress-Induced Cellular Senescence in Osteoporotic Bone Marrow Mesenchymal Stem Cells
Source: Oxid Med Cell Longev. 2022 Jan 18;2022:7420726. doi: 10.1155/2022/7420726 (PMC8789417; doi:10.1155/2022/7420726)
Supplement: Supplementary Materials — Supplementary Figure 1: evaluation of oxidative stress-induced premature senescence and apoptosis of sham and OVX BMMSCs. Supplementary Figure 2: the effects of melatonin on the cell cycle distribution and osteogenic differentiation of in H2O2-treated OVX BMMSCs. Supplementary Figure 3: the effect of melatonin on sham BMMSCs in the presence of H2O2-induced premature senescence. Supplementary Figure 4: melatonin improved the osteogenic differentiation of H2O2-treated sham BMMSCs. Supplementary Figure 5: intravenous injection of melatonin protected the trabecular bone micro-structure of OVX rats. Supplementary Figure 6: evaluation of the gene expression pattern of BMMSCs derived from melatonin-treated rats. Supplementary Figure 7: inhibition of SIRT1 by sirtinol aggravated premature senescence in OVX BMMSCs. Supplementary Figure 8: the effects of sirtinol on the trabecular bone microstructure of melatonin-treated OVX rats. Supplementary Figure 9: sirtinol treatment attenuated the antisenescence effect of melatonin on sham BMMSCs. Supplementary Figure 10: sirtinol treatment suppressed the osteogenic effect of melatonin on sham BMMSCs. [file 7420726.f1.docx]

**Supplementary Figures and Figure legends**


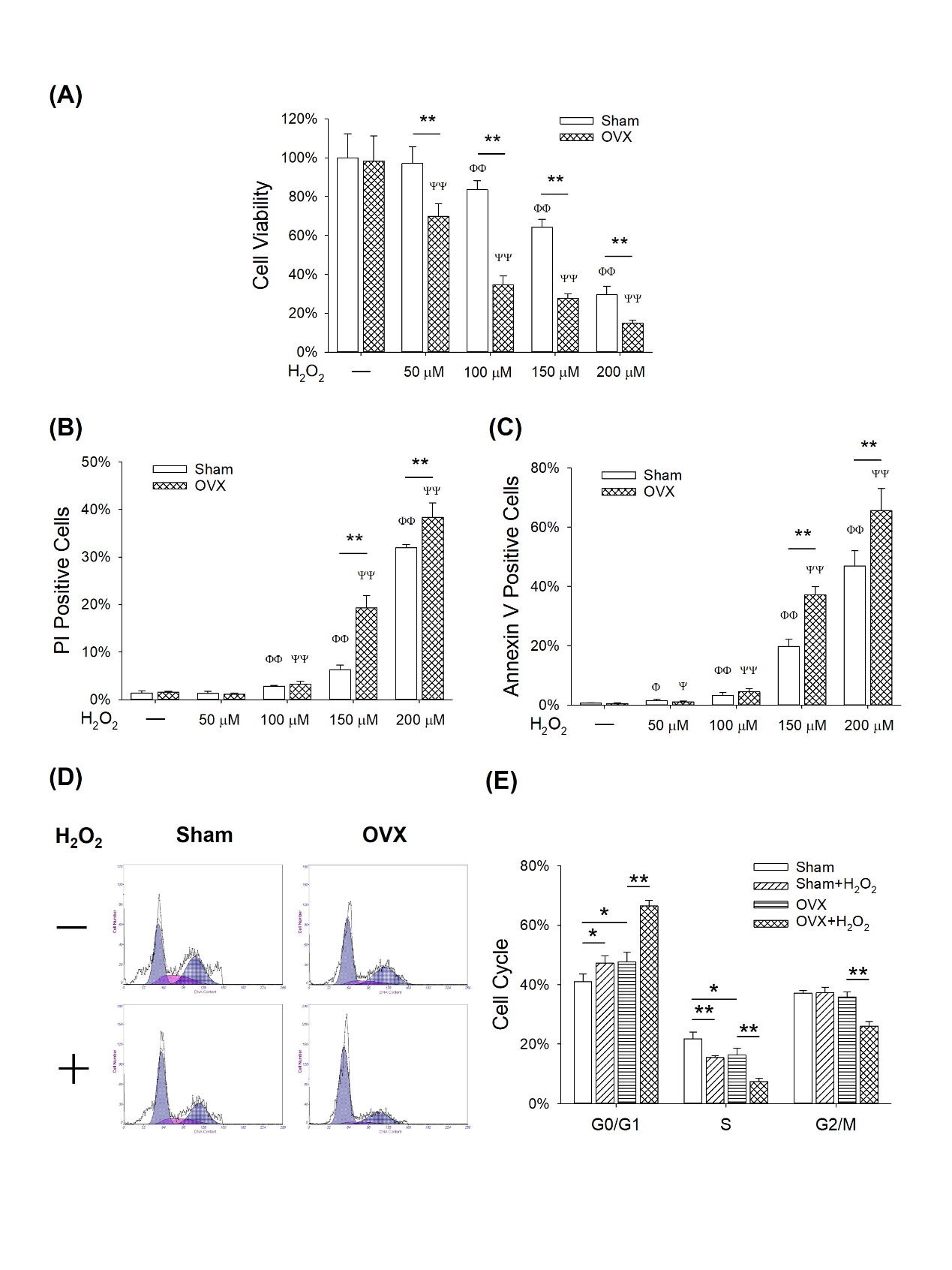


**Supplementary Fig. 1.** Evaluation of oxidative stress-induced premature senescence and apoptosis of Sham- and OVX BMMSCs. BMMSCs were exposed to H_2_O_2_ at 50 μM, 100 μM, 150 μM, and 200 μM for 2 h, after which cells were washed with fresh growth medium and cultured for additional 3 days. (A) The cell viability of Sham- and OVX-BMMSCs were determined using CCK-8 assays. (B-C) The effect of H_2_O_2_ treatments on cell apoptosis was analyzed by Annexin V and propidium iodide (PI) staining. (D-E) Analysis of cell cycle distribution showed that exposure to 100 μM of H_2_O_2_ induced a G0/G1 cell cycle arrest in OVX-BMMSCs. Values are presented as the mean ± S.E.M of eight independent experiments (*n* = 8) in cell viability assays, four independent experiments (*n* = 4) in cell apoptosis assays, three independent experiments (*n* = 3) in cell cycle assays. Statistically significant differences are indicated by * *p* < 0.05 or ** *p* < 0.01 between the indicated groups; ^Φ^ *p* < 0.05 or ^ΦΦ^*p* < 0.01 versus untreated Sham-BMMSCs; ^Ψ^ *p* < 0.05 or ^ΨΨ^ *p* < 0.01 versus untreated OVX-BMMSCs.


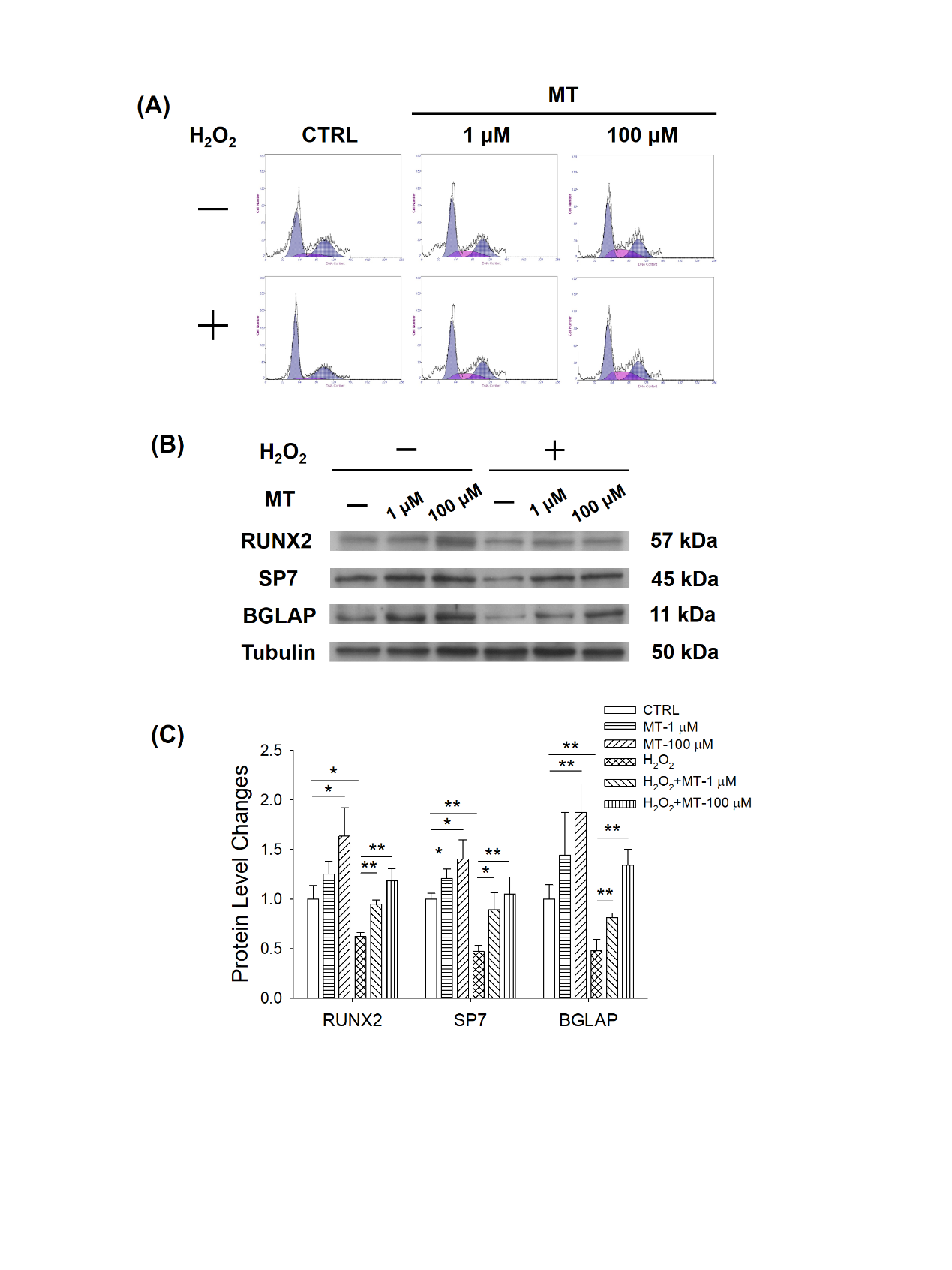


**Supplementary Fig. 2.** The effects of melatonin on the cell cycle distribution and osteogenic differentiation of in H_2_O_2_-treated OVX-BMMSCs. OVX-BMMSCs were first exposed to H_2_O_2_ (100 μM) for 2 h and then treated with melatonin (MT) at 1 μM and 100 μM concentrations for an additional 72 h. (A) The distribution of cell cycle in melatonin-treated and untreated OVX-BMMSCs was analyzed by flow cytometry. (B) Senescent OVX-BMMSCs were induced toward the osteogenic differentiation for 14 days. The protein levels of RUNX2, SP7, and BGLAP were determined using Western blot assays. (C) Quantification of the protein levels of RUNX2, SP7, and BGLAP. The values of these proteins were normalized to that of α-tubulin before comparison.Values are presented as the mean ± S.E.M of three independent experiments (*n* = 3) in Western blot assays. Statistically significant differences are indicated by * *p* < 0.05 or ** *p* < 0.01 between the indicated groups.


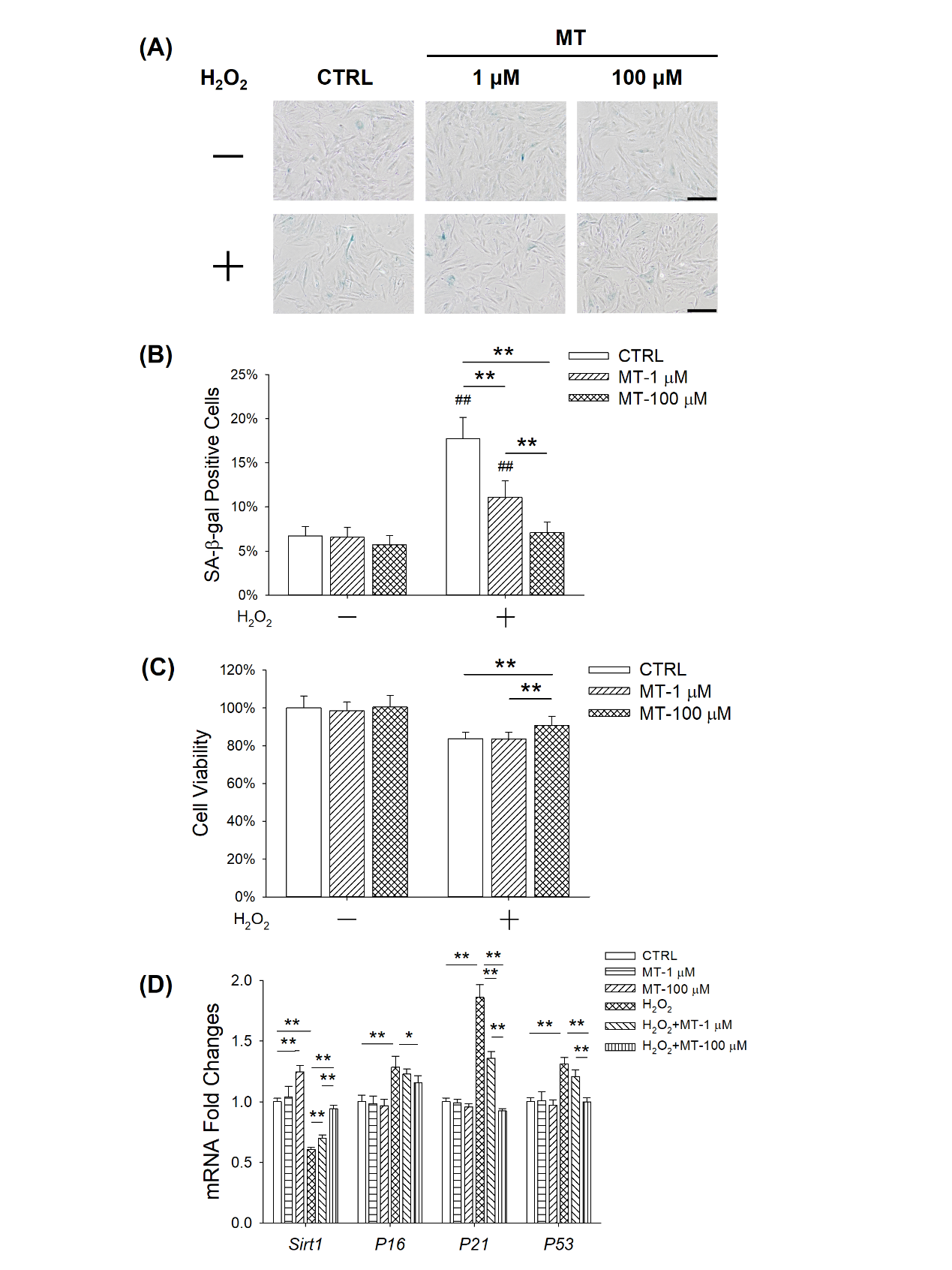


**Supplementary Fig. 3.** The effect of melatonin on Sham-BMMSCs in the presence of H_2_O_2_-induced premature senescence. BMMSCs were derived from sham-op rats and first exposed to H_2_O_2_ (100 μM) for 2 h and then treated with melatonin (MT) at 1 μM and 100 μM for an additional 72 h. (A-B) Senescent cells were labeled with senescence-associated β-galactosidase (SA-β-gal) staining. Scale bar = 100 μm. (C) The cell viability of Sham-BMMSCs was determined by CCK-8 assays. (D) The mRNA expressions of *Sirt1*, *P16*, *P21*, and *P53* were quantified. Values are presented as the mean ± S.E.M of six independent experiments (*n* = 6) in SA-β-gal staining, eight independent experiments (*n* = 8) in cell viability assays, and four independent experiments (*n* = 4) in RT-PCR experiments. Statistically significant differences are indicated by * *p* < 0.05 or ** *p* < 0.01 between the indicated groups. Statistically significant differences are indicated by * *p* < 0.05 or ** *p* < 0.01 between the indicated groups; ^#^ *p* < 0.05 or ^##^ *p* < 0.01 versus the CTRL group.


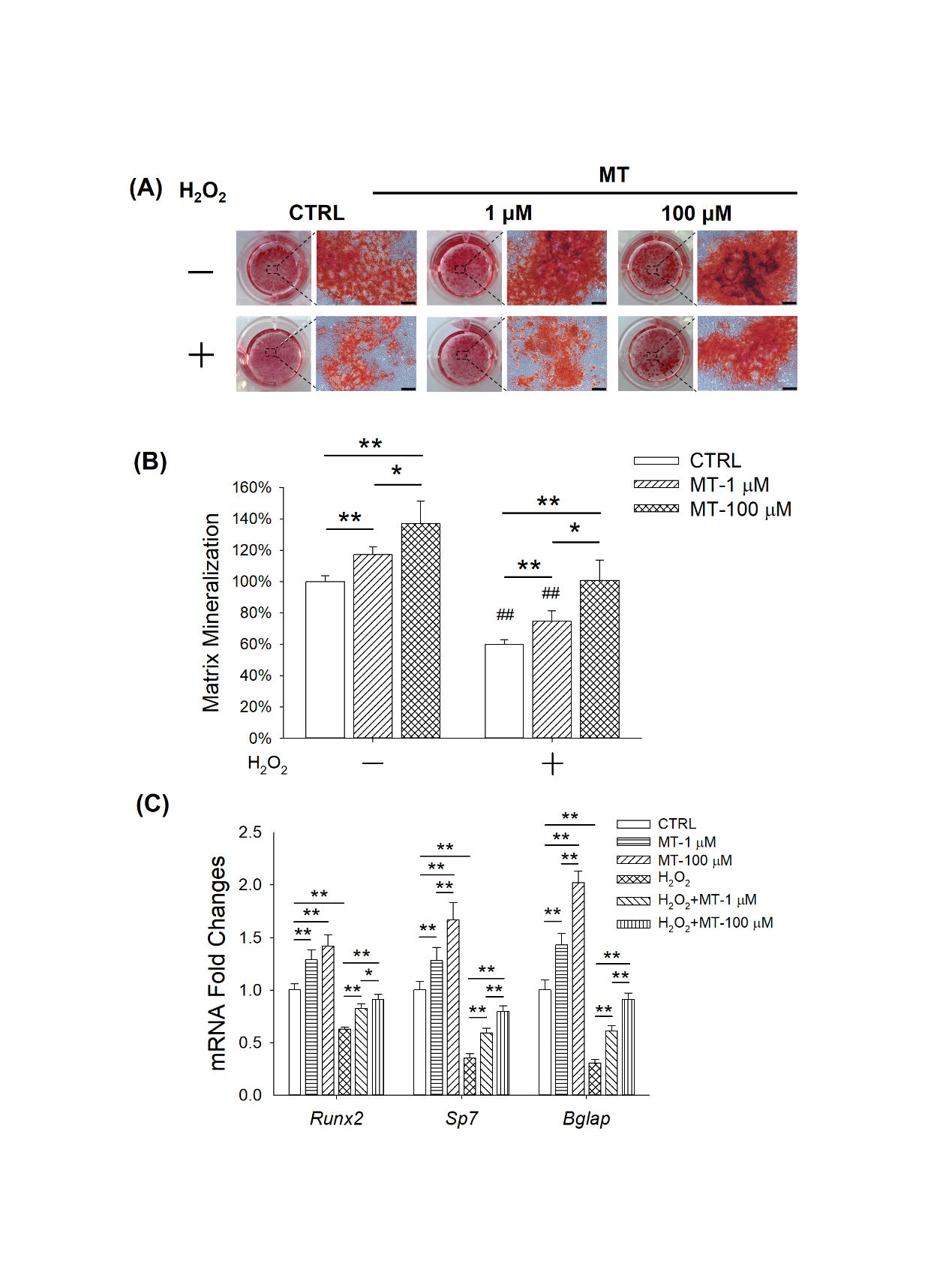


**Supplementary Fig. 4.** Melatonin improved the osteogenic differentiation of H_2_O_2_-treated Sham-BMMSCs. BMMSCs were derived from sham-op rats and first exposed to H_2_O_2_ (100 μM) for 2 h and were induced toward osteogenesis for 14 days with the supplement of melatonin (MT) at 1 μM and 100 μM. (A-B) Matrix mineralization was stained and quantified by Alizarin Red S (ARS). Scale bar = 200 μm. The values were normalized to those of the untreated Sham-BMMSCs. (C) The mRNA levels of osteoblast-specific marker genes, including *Runx2*, *Sp7*, and *Bglap* were quantified with real-time RT-PCR using *Gapdh* as the reference gene for normalization. Values are presented as the mean ± S.E.M of four independent experiments (*n* = 4) in ARS assays and four independent experiments (*n* = 4) in RT-PCR experiments. Statistically significant differences are indicated by * *p* < 0.05 or ** *p* < 0.01 between the indicated groups. Statistically significant differences are indicated by * *p* < 0.05 or ** *p* < 0.01 between the indicated groups; ^#^ *p* < 0.05 or ^##^ *p* < 0.01 versus the CTRL group.


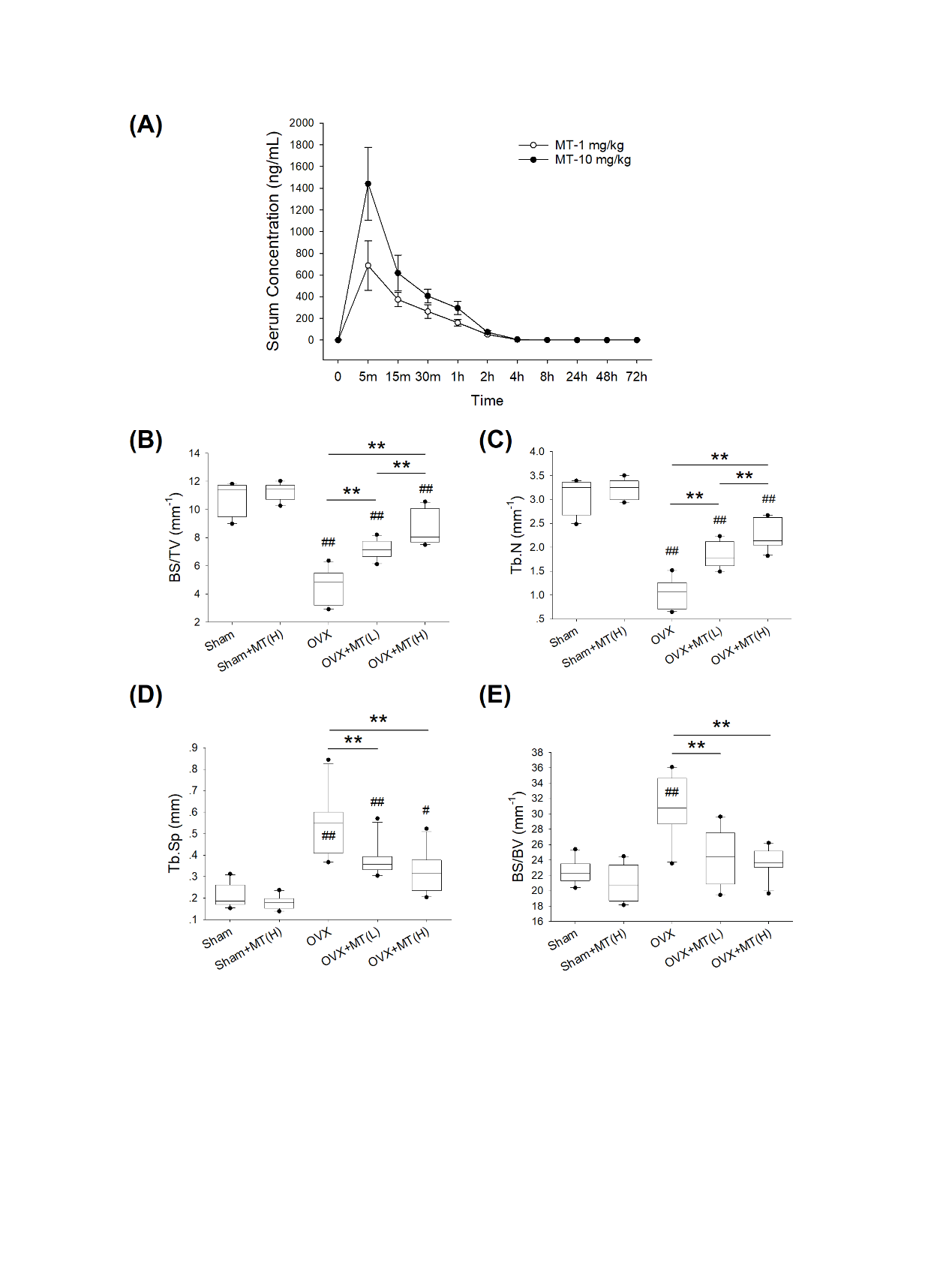


**Supplementary Fig. 5.** Intravenous injection of melatonin protected the trabecular bone micro-structure of OVX rats. After ovariectomy, melatonin (MT) was injected into OVX rats through the tail vein at a low dosage [1 mg/kg, OVX+MT(L)] or a high dosage [10 mg/kg, OVX+MT(H)], while Sham-op rats were injected with melatonin at 10 mg/kg [Sham+MT(H)]. The rats of Sham and OVX groups received saline containing the same amount of ethanol. (A) After intravenous administration with melatonin at two doses (1 or 10 mg/kg), melatonin concentration in serum (ng/mL) was measured at each predefined time point. (B-E) Micro-CT and 3D reconstruction were used to histomorphometrically analyze the rat femurs. The BS/TV (mm^-1^) (B), trabecular number (Tb.N., mm^-1^) (C), trabecular separation (Tb.Sp., mm) (D), and BS/BV (mm^-1^) (E) of the rat femurs were analyzed using the μCT system. Values are presented as the mean ± S.E.M of ten samples in each group (*n* = 10) in micro-CT and 3D reconstruction assays. Statistically significant differences are indicated by * *p* < 0.05 or ** *p* < 0.01 between the indicated groups; ^#^ *p* < 0.05 or ^##^ *p* < 0.01 versus the Sham group.


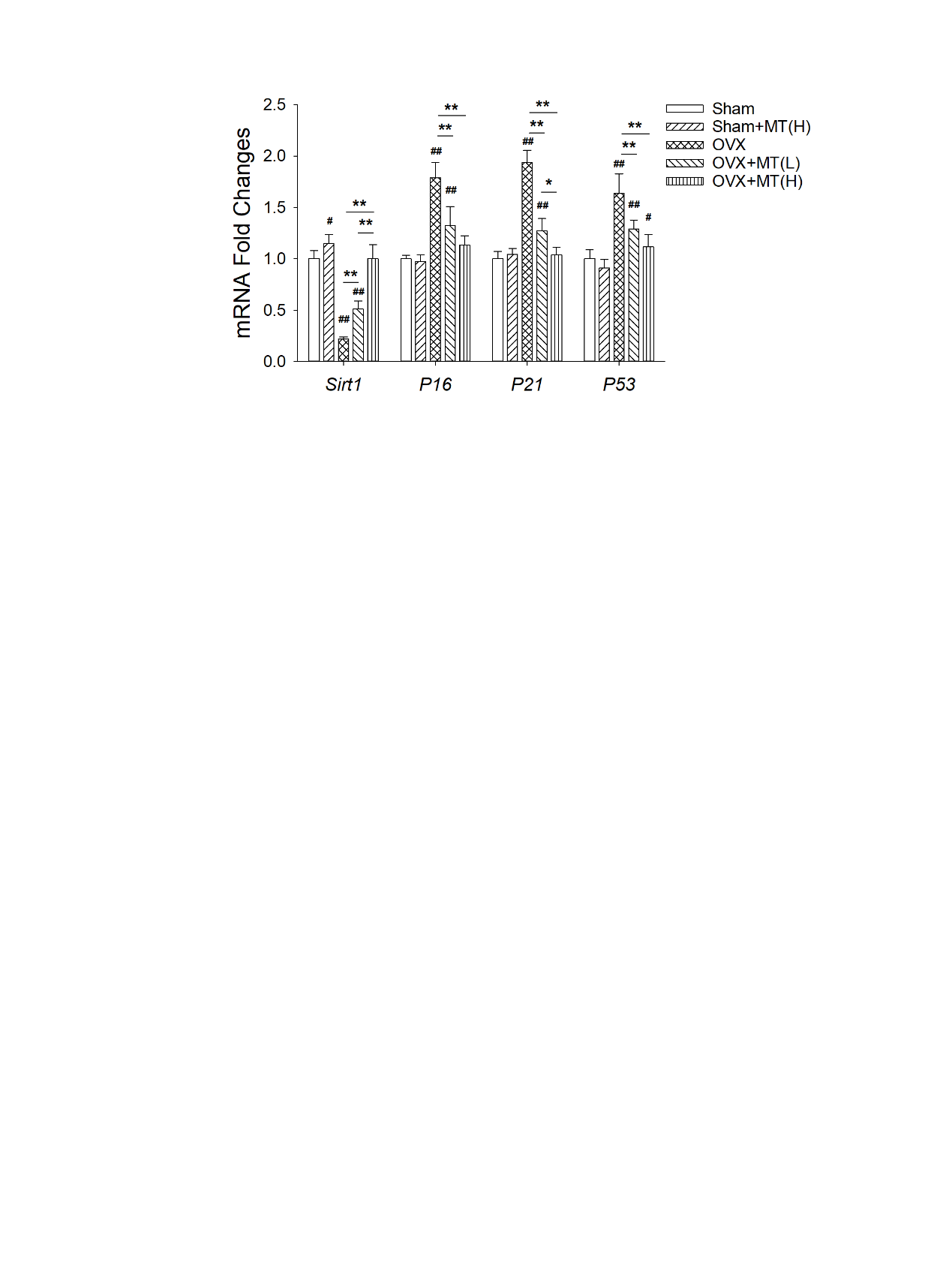


**Supplementary Fig. 6.** Evaluation of the gene expression pattern of BMMSCs derived from melatonin-treated rats. BMMSCs were isolated from untreated and melatonin-treated Sham or OVX rats and then exposed to H_2_O_2_ for 2 h. The mRNA expressions of *Sirt1*, *P16*, *P21*, and *P53* were quantified using real-time RT-PCR. Values are presented as the mean ± S.E.M of four independent experiments (*n* = 4) in RT-PCR experiments. Statistically significant differences are indicated by * *p* < 0.05 or ** *p* < 0.01 between the indicated groups. Statistically significant differences are indicated by * *p* < 0.05 or ** *p* < 0.01 between the indicated groups; ^#^ *p* < 0.05 or ^##^ *p* < 0.01 versus the Sham group.


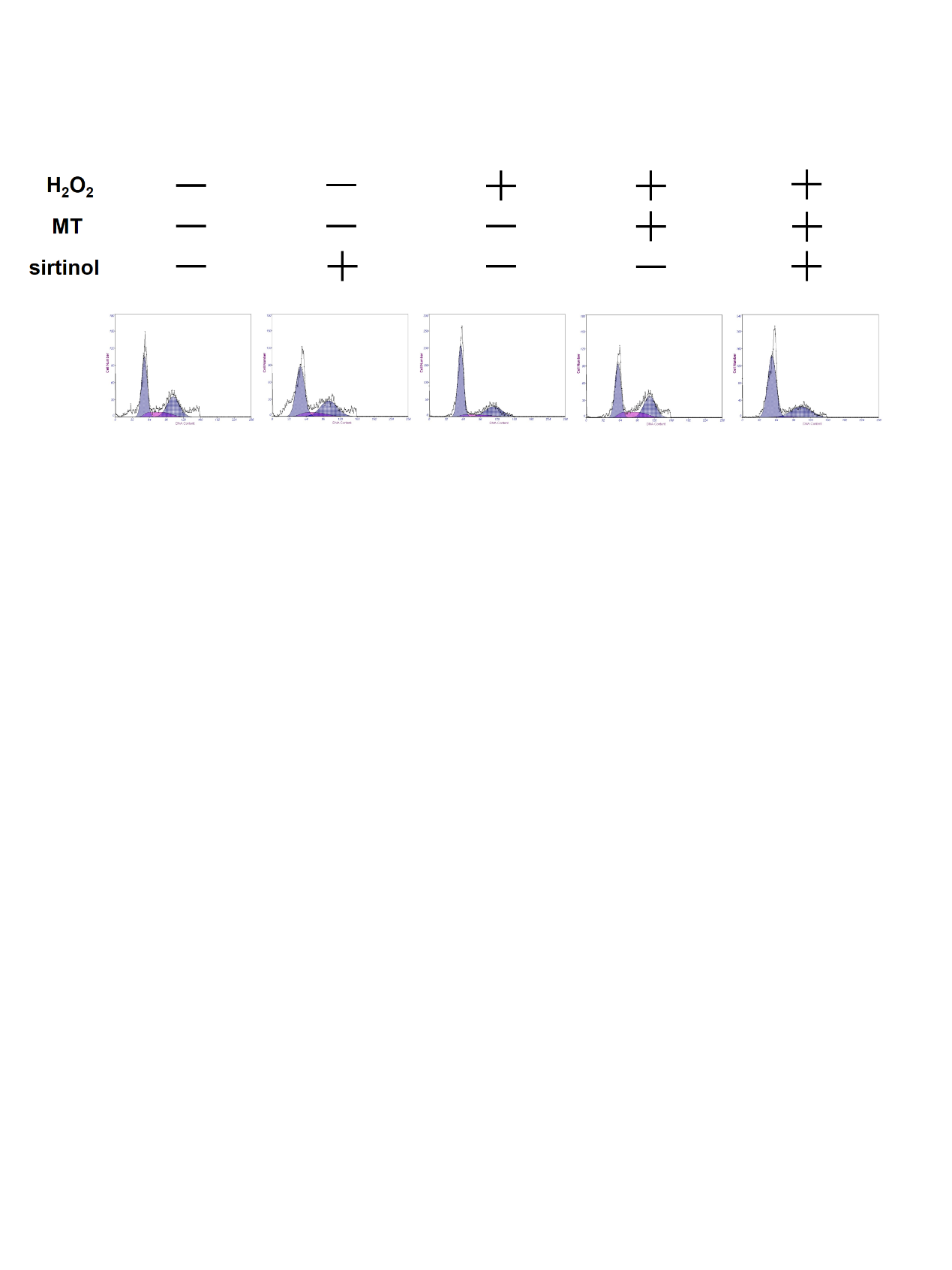


**Supplementary Fig. 7.** Inhibition of SIRT1 by sirtinol aggravated premature senescence in OVX-BMMSCs. Cells were first exposed to H_2_O_2_ for 2 h and then treated with 100 μM of melatonin (MT) with or without 40 μM of sirtinol for 72 h. The effect of sirtinol treatment on the distribution of cell cycle was determined by flow cytometry.


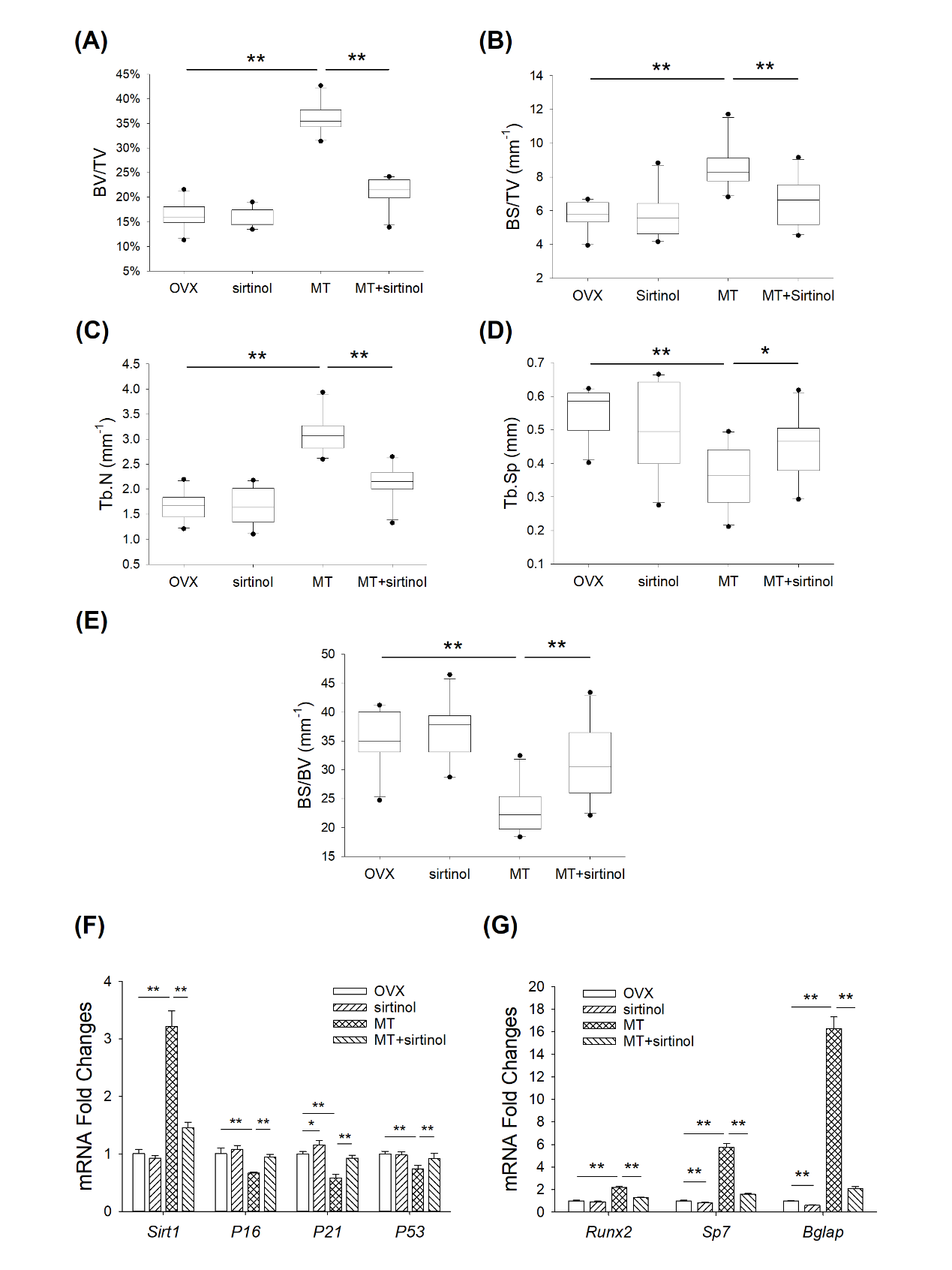


**Supplementary Fig. 8.** The effects of sirtinol on the trabecular bone micro-structure of melatonin-treated OVX rats. OVX rats were injected with melatonin (MT, 10 mg/kg) or sirtinol (1 mg/kg). Micro-CT and 3D reconstruction were used to analyze the trabecular bone micro-structure of the melatonin- or sirtinol-treated OVX rats. The BV/TV (A), BS/TV (mm^-1^) (B), trabecular number (Tb.N., mm^-1^) (C), trabecular separation (Tb.Sp., mm) (D), and BS/BV (mm^-1^) (E) of the rat femurs were analyzed. (F) BMMSCs were isolated from melatonin- and sirtinol-treated OVX rats, exposed to H_2_O_2_ (100 μM) for 2 h, and cultured for an additional 72 h. The mRNA expressions of *Sirt1*, *P16*, *P21*, and *P53* were quantified using real-time RT-PCR. (G) BMMSCs derived from melatonin- or sirtinol-treated OVX rats were induced to undergo osteogenic differentiation. The mRNA levels of osteoblast-specific marker genes, *Runx2*, *Sp7*, and *Bglap* were quantified with real-time RT-PCR in which *Gapdh* was used for normalization. Values are presented as the mean ± S.E.M of ten samples in each group (*n* = 10) in micro-CT and 3D reconstruction assays and four independent experiments (*n* = 4) in RT-PCR experiments. Statistically significant differences are indicated by * *p* < 0.05 or ** *p* < 0.01 between the indicated groups.


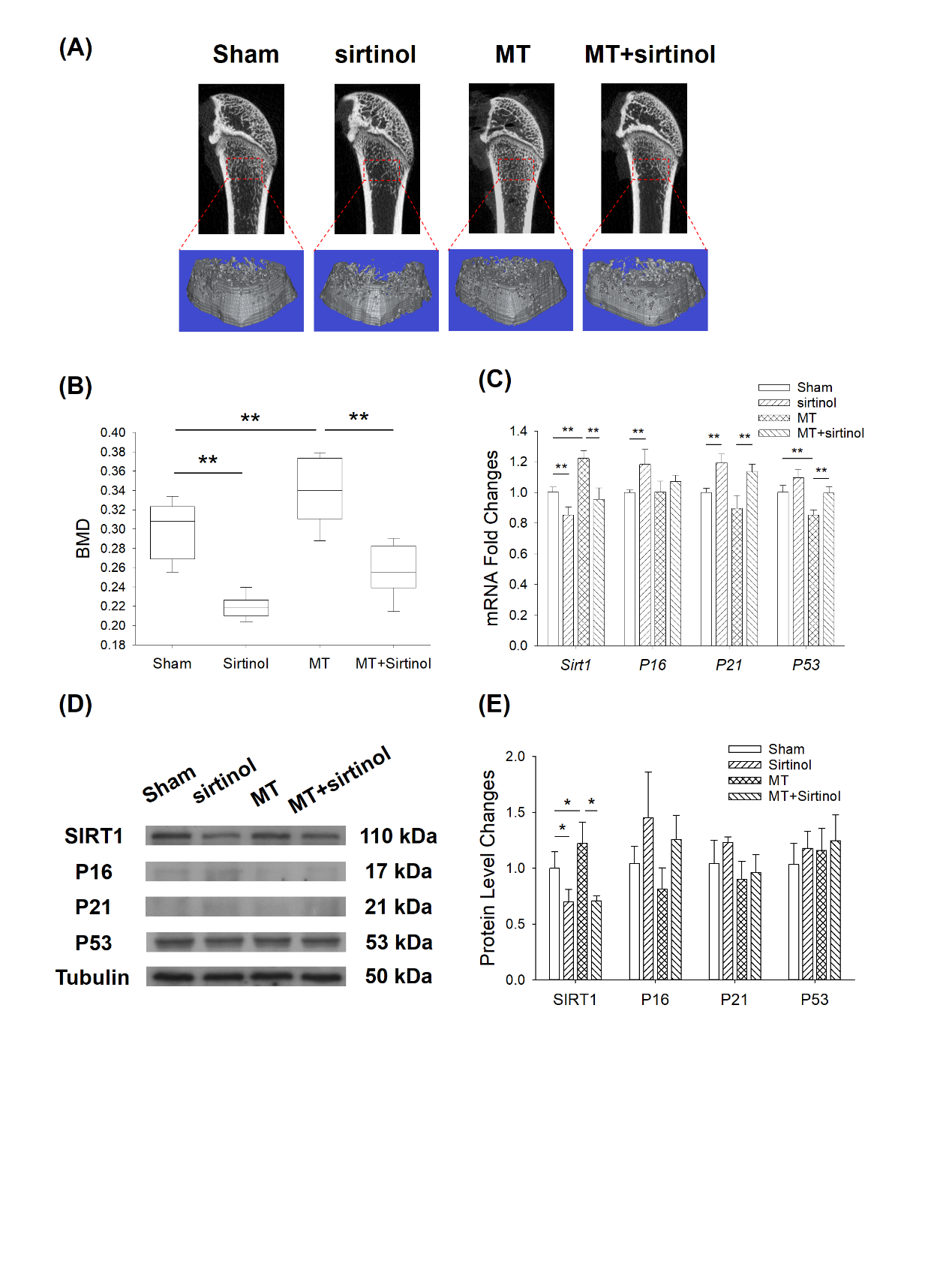


**Supplementary Fig. 9.** Sirtinol treatment attenuated the anti-senescence effect of melatonin on Sham BMMSCs. Sham-op rats were injected with melatonin (MT, 10 mg/kg) or sirtinol (1 mg/kg) through the tail vein. (A) Micro-CT and 3D reconstruction were used to analyze the trabecular bone microstructure. (B) The effect of sirtinol treatment on the values of bone mineral density (BMD). (C) BMMSCs were isolated from melatonin- and sirtinol-treated Sham rats, exposed to H_2_O_2_ (100 μM) for 2 h, and cultured for an additional 72 h. The mRNA expressions of *Sirt1*, *P16*, *P21*, and *P53* were quantified using real-time RT-PCR. (D-E) The protein levels of SIRT1, P16, P21, and P53 were determined using Western blot assays. The values of these proteins were normalized to that of α-tubulin before comparison. Values are presented as the mean ± S.E.M of six samples in each group (*n* = 6) in micro-CT and 3D reconstruction assays, four independent experiments (*n* = 4) in RT-PCR experiments and three independent experiments (*n* = 3) in Western blot assays. Statistically significant differences are indicated by * *p* < 0.05 or ** *p* < 0.01 between the indicated groups.


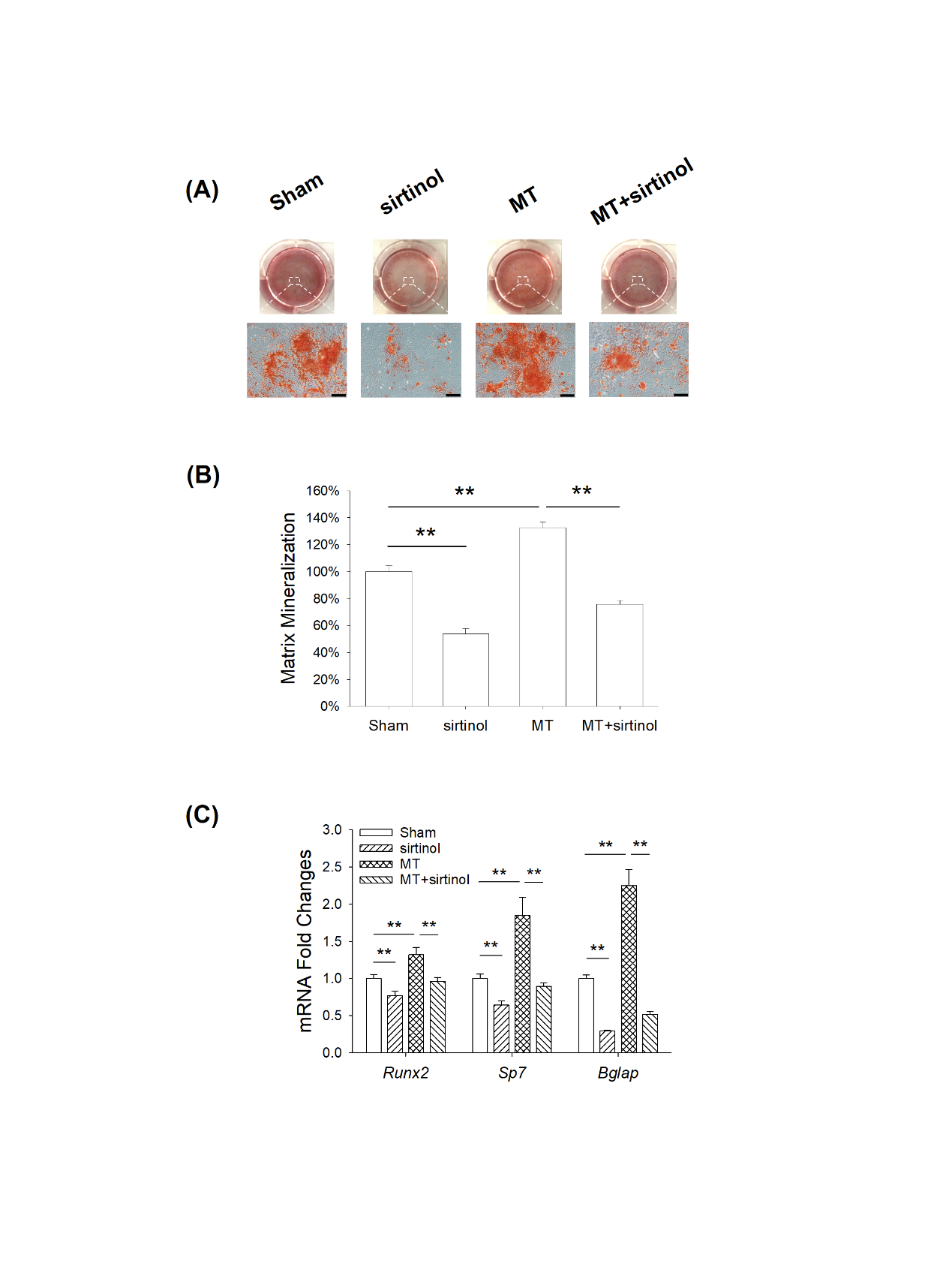


**Supplementary Fig. 10.** Sirtinol treatment suppressed the osteogenic effect of melatonin on Sham BMMSCs. Sham-op rats were injected with melatonin (MT, 10 mg/kg) or sirtinol (1 mg/kg). BMMSCs were isolated from melatonin- and sirtinol-treated Sham rats, and exposed to H_2_O_2_ (100 μM) for 2 h. The cells were induced toward osteogenic differentiation for 14 days. (A-B) Matrix mineralization was assessed by Alizarin Red S (ARS) staining. Scale bar = 200 μm. The values shown were normalized to those of the Sham group. (C) The mRNA levels of osteoblast-specific marker genes, *Runx2*, *Sp7*, and *Bglap* were quantified with real-time RT-PCR in which *Gapdh* was used for normalization. Values are presented as the mean ± S.E.M of four independent experiments (*n* = 4) in ARS assays and four independent experiments (*n* = 4) in RT-PCR experiments. Statistically significant differences are indicated by * *p* < 0.05 or ** *p* < 0.01 between the indicated groups.
